# Modeling Heterogeneity in Health Risk Behaviors With a Dynamic Mixture Model Informed by Textual Occupational Data

**DOI:** 10.1002/sim.70691

**Published:** 2026-08-10

**Authors:** Lorenzo Schiavon, Mattia Stival, Angela Andreella, Stefano Campostrini

**Affiliations:** ^1^ Department of Statistical Sciences University of Padova Padova Italy; ^2^ Department of Mathematics University of Padova Padova Italy; ^3^ Department of Economics Ca' Foscari University of Venice Venice Italy

**Keywords:** Bayesian mixture of ordinal probit models, behavioral risk‐factor surveillance, epidemiology, observational health studies, sequential Monte Carlo, structural topic modeling

## Abstract

Health risk behaviors, including smoking, poor nutrition, alcohol misuse, and physical inactivity (SNAP), are leading contributors to chronic disease burden and healthcare costs worldwide. Their prevalence is shaped not only by individual demographic characteristics but also by contextual factors such as socioeconomic and occupational environments. We develop a Bayesian topic‐informed dynamic mixture model to analyze SNAP behaviors using data from the Italian health and behavioral surveillance system PASSI. Free‐text occupational descriptions are integrated through structural topic modeling to define latent occupational groups that inform the mixture weights of a multivariate ordered probit model. Covariate effects are allowed to vary across occupational clusters and evolve over time. To enhance interpretability and facilitate variable selection, we incorporate non‐local spike‐and‐slab priors on regression coefficients. We further develop a tailored online learning strategy based on sequential Monte Carlo, enabling efficient updating of inference as new surveillance data become available. The proposed approach provides a flexible and interpretable framework for monitoring how occupational contexts modulate the impact of socio‐demographic factors on multiple health risk behaviors over time, supporting targeted public health interventions. More broadly, the methodology is applicable to other longitudinal health surveillance and clinical settings where complex contextual information is available in textual form.

**Abbreviations**
HMC: Hamiltonian Monte Carlo
SMC: sequential Monte Carlo
SNAP: smoking, poor nutrition, alcoholmisuse, physical inactivity
STM: structural topic modeling

## 1 Introduction

Health risk behaviors such as smoking, poor nutrition, alcohol misuse, and physical inactivity, collectively referred to as SNAP, are major contributors to chronic diseases and global mortality [1]. These behaviors not only compromise individual health but also significantly burden healthcare systems [2]. In Italy, Possenti et al. [3] estimated that smoking was responsible for one‐third of hospitalizations among adults over 30 in 2018, costing €1.64 billion, while obesity‐related costs reached €13.34 billion in 2020 [4]. In a review of 26 studies, Manthey et al. [5] estimated that alcohol use alone accounts for approximately 1.5% of GDP. Understanding how risk factors emerge and coalesce is key to designing effective, targeted interventions to impact public health outcomes [6], while this poses substantial statistical challenges, particularly in large‐scale surveillance data.

SNAP behaviors are known to correlate with demographic variables such as age, sex, and geographical location [7, 8]. Thus, modeling individual propensity to SNAP through appropriate regression techniques helps explain population variability in SNAP levels, collected in the matrix Y, based on demographic covariates X [9, 10].

Beyond macro‐environmental influences, risk behaviors are often most affected by the socioeconomic micro‐environment in which an individual lives, such that we expect a heterogeneous effect B of the demographic characteristic X across different environments. In particular, the occupational environment, along with socioeconomic status, plays a key role in shaping a person's social and economic context. In adulthood, occupation not only determines time use and exposure to stressors but also represents a primary source of social interactions and lifestyle norms, thereby shaping individual habits [11, 12].

We analyzed the occurrence of SNAP behaviors in PASSI data [13], a monthly‐based sample survey that gathers information on behavioral risk factors, socio‐demographic characteristics, and self‐reported non‐communicable diseases in Italy. Using these data to understand the link between occupation and behavioral risk factors may inform targeted public health policies. While job strain is often studied as a driver of unhealthy behaviors [14], broader associations between SNAP factors and job type remain understudied. Existing research typically focuses on specific professions [15], behaviors like alcohol or smoking [16, 17, 18], employment status [19, 20], or age groups [21]. However, an integrated understanding of how multiple risk factor behaviors depend on both socioeconomic and occupational context, demographic drivers, and temporal trends is still lacking, often due to data limitations. These are overcome in this study since we use the PASSI surveillance system, which offers rich information on both socio‐demographic characteristics and lifestyle habits of each respondent. In addition, PASSI collects occupational data through both an open‐ended employment question and the ISTAT occupation classification and sectors. We assume that integrating occupational information together with socioeconomic traits can provide a more comprehensive representation of an individual's social micro‐environment.

For each respondent n, we define a vector of words $w_{n}$ obtained by combining the open‐ended employment response with the textual descriptions from the ISTAT classification and occupational sectors. We also define a vector $v_{n}$ containing socioeconomic indicators. To enable a quantitative, data‐driven characterization of occupations while preserving the richness of this information, we employ topic modeling techniques that incorporate exogenous covariates. These methods summarize the textual and socioeconomic information into homogeneous clusters of individuals, with $s_{n}$ indicating the cluster membership of respondent n. We expect these clusters to reflect both the job description W and groups of individuals characterized by different relationships B between demographic factors and SNAP, as illustrated in the model scheme reported in Figure 2. Mixture models provide a natural framework for this purpose, capturing unobserved heterogeneity and reducing the dimensionality of complex population structures [22]. However, in this setting, an uninformed mixture would only capture latent patterns from the SNAP outcomes, failing to exploit available occupational and socioeconomic variables to characterize this variation and anchor the components to interpretable micro‐environments. Alternatively, one could inform the mixture weights using standardized occupational and socioeconomic variables. However, a fully crossed classification based on these variables would generate sparse, high‐dimensional partitions. We therefore propose a novel approach where free‐text occupational information is carefully summarized into meaningful latent topics that inform prior probabilities for $s_{n}$, which the SNAP model then updates using observed health behaviors. Unlike existing applications in public health [23], our approach uses the topic model as an informative prior rather than the final clustering target.

Following societal shifts, the influence of social and demographic factors on risky health behaviors evolves over time [24]. For example, although such behaviors were traditionally more prevalent among men, decreasing gender inequalities and shifting social norms have led to more balanced patterns across sexes [25]. These evolving associations highlight the need for a time‐varying coefficient matrix $B^{\left(t\right)}$ where t=1,…,T. These effects are expected to vary across occupational and socioeconomic groups, reflecting differences in education and changes in work environments. Technological automation has increased sedentary behavior in manual labor jobs [26], while the COVID‐19 pandemic accelerated the adoption of remote work, particularly in higher‐educated and higher‐skilled sectors. This latter shift has altered daily routines and health behaviors such as diet and physical activity, with uneven impacts across socioeconomic and occupational groups [27]. Figure 1 illustrates short‐term variation in risk trajectories for three job categories among those defined by ISTAT [28]. These trends motivate a model in which regression coefficients vary over time and across latent micro‐environment groups, and where parameters can be updated continuously as new data become available. Dynamic mixtures are challenging because they require joint inference on latent memberships, group‐specific temporal effects, and newly arriving observations. Repeated static mixtures would ignore temporal dependence (as discussed in Blei and Lafferty [29]), whereas fully joint dynamic mixture estimation can be computationally costly in large surveillance systems [30, 31]. We address these issues by developing an online Bayesian learning strategy that sequentially updates both group allocations and time‐varying regression coefficients as new data become available. This yields an interpretable and flexible framework tailored to large‐scale surveillance data.

**FIGURE 1:**
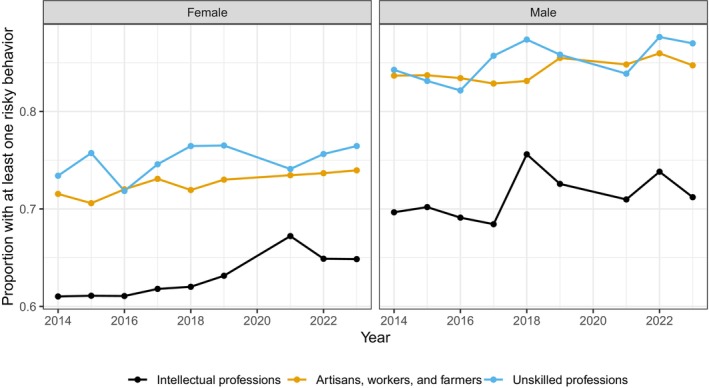
Proportions of individuals with at least one risky behavior are reported by sex, occupation, and year.

Similar challenges often arise in biomedical and clinical research, where investigators may require statistical models that jointly analyze multiple correlated ordinal outcomes [32], such as disease onset or severity, while allowing covariate effects to evolve over time and differ across latent patient subgroups informed by unstructured textual information, including clinical notes or previously reported findings in the literature [33].

We summarize our modeling goals as follows. A suitable statistical framework should: (1) model the relationship between demographic variables $x_{n}$ and SNAP outcomes $y_{n}$ as varying across time t and latent groups $s_{n}\in \{1,\ldots ,K\}$ to capture heterogeneity; (2) incorporate textual occupational descriptions $w_{n}$ and socioeconomic indicators $v_{n}$ through structured topic modeling to inform group membership; (3) support online parameter updates as new data become available; and (4) enforce sparsity in the effects, since not all covariates are expected to influence SNAP outcomes in every group or time period. To meet these goals, we introduce a dynamic mixture of multivariate ordered probit models, where the latent outcome is centered at $x_{n}B_{k}^{\left(t\right)}$, with $B_{k}^{\left(t\right)}$ denoting the group‐ and time‐specific coefficient matrix. The proposed framework aims to offer a novel perspective by explicitly embracing the inherent complexity of the data under analysis [34].

The manuscript is organized as follows. Section 2 presents the proposed dynamic mixture model for multivariate ordinal health outcomes, in which the structural topic model (STM) [35] is used to incorporate textual occupational and socioeconomic information into the mixture weights. Sparsity and temporal dynamics in $B_{k}^{\left(t\right)}$ are modeled via a spike‐and‐slab prior with a non‐local slab formulation [36]. Section 3 introduces a newly developed sequential Monte Carlo (SMC)‐based online learning algorithm, assessed through simulation studies in Section 4. Section 5 applies the model to data from the Italian health surveillance system PASSI [13], illustrating its potential to support the analysis of evolving health outcomes, followed by discussion and conclusions in Section 6.

## 2 A Free‐Text‐Informed Dynamic Mixture Model for Ordinal Health Outcomes

### 2.1 Model Specification

In this subsection, we present the general framework of a dynamic mixture of multivariate ordered probit models for health‐related behaviors. The incorporation of occupational and socioeconomic information through a STM, as well as the dynamic shrinkage prior for the regression coefficients, are described in the subsequent subsections.

Let $y_{n}=[y_{n1},\ldots ,y_{nP}]$ denote the response vector for respondent n, where the pth entry $y_{np}=c+1$ indicates the ordinal level c of the risky behavior p∈{1,…,P} for respondent n∈{1,…,N}, with $c\in \{0,\ldots ,C_{p}-1\}$ and $C_{p}$ denoting the number of distinct categories for risky behavior p. Without loss of generality, the marginal ordinal variable $y_{np}$ can always be represented as

$$y_{np}=\overset{C_{p-1}}{\underset{c=0}{\sum }}1(z_{np}>\tau_{pc}),$$

in terms of a latent variable $z_{np}∼F$ where F is a suitable distribution, and threshold $\tau_{pc}$ is defined such that $\tau_{p(c-1)}<\tau_{pc}\in \mathbb{R}$ for $c\in \{1,\ldots ,C_{p}-1\}$ and $\tau_{p0}=-\infty$, $\tau_{pC_{p}}=\infty$ for any p=1,…,P. Common models such as ordered probit [37] and logistic [38] regressions exploit this representation by assuming F belongs to the Gaussian or logistic distribution families, respectively.

For each respondent, we are interested in exploring the relationship between the SNAP level $y_{n}$ and their demographic profile described by the covariate vector $x_{n}=[x_{n1},\ldots ,x_{nQ}]$, including characteristics as age class, sex, and the Italian macro‐region of residence. In this notation, $x_{nq}$ denotes the qth covariate for subject n. As previously introduced, we expect this relationship to vary across the population.

To account for such heterogeneity, we model the vector $y_{n}$ using a mixture of K multivariate ordered probit regression models

(1) $$y_{np}=\overset{C_{p-1}}{\underset{c=0}{\sum }}1(z_{np}>\tau_{pc}),\;\left(z_{n}|x_{n},s_{n}=k,B_{k}^{\left(t\right)}\right)∼{𝒩}_{P}(x_{n}B_{k}^{\left(t\right)},I_{P}),$$

where $z_{n}=[z_{n1},\ldots ,z_{nP}]$ is a P‐variate latent Gaussian vector, conditional on $s_{n}$, which identifies the latent mixture component for respondent n. $x_{n}B_{k}^{\left(t\right)}$ is then the mean of $z_{n}$, given the respondent's group allocation $s_{n}=k$ where k∈{1,…,K} and its associated matrix of regression coefficients $B_{k}^{\left(t\right)}\in {\mathbb{R}}^{Q\times P}$. Notably, the matrix of coefficients is indexed by (t), indicating the time instant (month) at which data are collected, reflecting dynamic patterns within and between the years. For simplicity in notation, this time indexing is not reported on the quantities indexed by the respondent's index n, implicitly referring to n also to the time $\left(t_{n}\right)$ at which the data were collected.

The mixture formulation explores whether the propensity for risky behaviors, conditional on demographic characteristics, varies across the population. We expect this heterogeneity to be driven by the context in which individuals live. Thus, if additional information is available, this can be used to model the prior knowledge about the group membership of the individuals. In our framework, both occupational text $w_{n}$ and socioeconomic characteristics $v_{n}$ jointly inform group membership $s_{n}$ through the STM, defining a unified socioeconomic construct that captures occupational micro‐environments. Socioeconomic indicators are incorporated at this stage rather than directly in the SNAP regressions, thereby avoiding redundancy. Figure 2 illustrates the relationships among all the components described above, highlighting how the respondent's group allocation $s_{n}=k$ links the topic model to the mixture of probit regression models.

**FIGURE 2:**
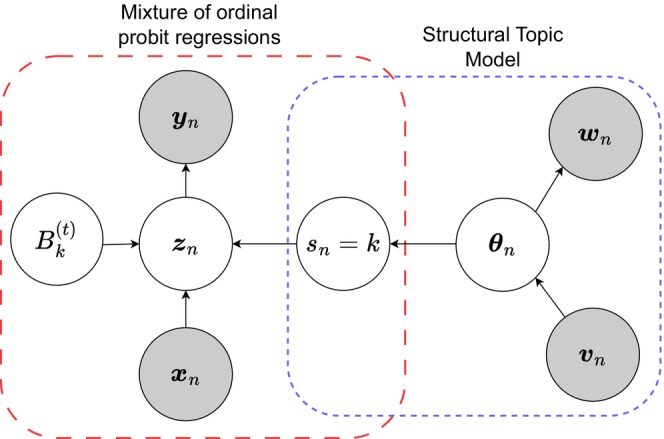
Model diagram describing the role of $s_{n}$: A bridge between the topic model and the mixture of probit regression models. Observed data vectors are colored in grey.

### 2.2 Topic Modeling Informed Prior for Group Membership

The occupational environment, often reflecting an individual's broader socioeconomic context, plays a key role in shaping health behaviors [11, 12]. Unlike other contextual factors, such as family or community setting, occupational information is routinely collected in health surveillance systems like PASSI.

In light of this, we hypothesize the existence of latent clusters representing distinct socioeconomic and occupational groups, which influence individuals' propensity to engage in risky behaviors. We then model the prior distribution of the mixture weights, $\mathrm{Pr}(s_{n}=k)$ with k∈{1,…,K}, by incorporating occupational information. We assume that respondents with similar occupations, particularly in socioeconomic terms, are more likely to be assigned to the same mixture component. However, open‐ended responses may be incomplete or vague, while relying solely on coded classifications could produce an excessive number of small, non‐representative categories. Thus, we link the textual description of the occupation of one respondent to its latent representation $s_{n}$, which defines, in our setting, both the occupational group and the a priori behavioral group. To enhance the grouping of textual information, we also leverage individual socioeconomic indicators, that is, education level and economic situation, stored in the N×U matrix V.

To integrate the socioeconomic data in the topic modeling specification, we employ the STM proposed by Roberts et al. [35], which allows the inclusion of exogenous covariates. Consistent with the authors, we specify

(2) $$\begin{array}{c} s_{n}|\theta_{n}∼{\text{Multinomial}}_{K}(1,\theta_{n}),\;\theta_{n}∼{\text{LogisticNormal}}_{K-1}(\Lambda^{⊤}v_{n},\Psi ), \end{array}$$

where $\theta_{n}$ is a K‐dimensional vector defined on the simplex ${\left(0,1\right)}^{K}$. Here, $v_{n}$ denotes the nth row of the covariate matrix V, while Λ is a (K−1)×U coefficient matrix and Ψ is a positive semi‐definite (K−1)×(K−1) covariance matrix, both treated as hyperparameters.

For the textual information, the data‐generating process is then completed by defining a suitable distribution over the H words in the vocabulary of the textual collection. The ith word $w_{ni}$ in the text of subject n is sampled as:

(3) $$w_{ni}|\zeta_{ni}∼{\text{Multinomial}}_{H}(1,\gamma_{\zeta_{ni}}),\;\zeta_{ni}|\theta_{n}∼{\text{Multinomial}}_{K}(1,\theta_{n}),$$

where $\gamma_{k}$ are cluster‐specific hyperparameter vectors set on the simplex ${\left(0,1\right)}^{H}$. For further details on the topic‐modeling specification, we refer to Roberts et al. [35]. Note that, in our specific framework, the topic proportion vector $\theta_{n}$ plays a dual role.

On one hand, it defines the probability distribution of topic $\zeta_{nh}$ for each word within the text of respondent n; then, conditional on $\zeta_{ni}$, a word is selected from the corresponding distribution, that is, Equation (2). On the other hand, being $\theta_{n}$ also the probability of the latent occupational group $s_{n}$, it also represents the a priori behavioral group, that is, Equation (3). Suppose no data on SNAP behaviors are observed. In that case, the posterior inference on $\theta_{n}$ given the texts can be achieved by considering the STM implementation in the R package stm [39]. By exploiting variational techniques, approximated posterior inference can be quickly conducted even with large datasets.

Notably, the cluster allocation $s_{n}$ is the only term of STM that is involved in the mixture of probit regression models. As later explained in Section 3, this enables us to perform two‐stage modular inference by sequentially estimating the STM and adjusting the estimates based on behavioral data.

### 2.3 Dynamic Nonlocal Prior for Regression Coefficients

When considering the regression parameters, we expect two main features. First, certain risky behaviors within occupational groups are likely to evolve over time. Second, among all regression parameters, only a subset is expected to have a meaningful impact, while many effects are negligible. To capture both aspects, we specify a dynamic prior that induces sparsity in the regression coefficients.

To model temporal dependence, we define a time‐dynamic process for each entry $\beta_{kpq}^{\left(t\right)}$ of $B_{k}^{\left(t\right)}$, representing the effect at time t of the qth covariate in X on outcome p for group k. We assume a Markovian dependence structure, that is, $p(\beta_{kpq}^{\left(t\right)}|\beta_{kpq}^{\left(1\right)},\ldots ,\beta_{kpq}^{\left(t-1\right)})=p(\beta_{kpq}^{\left(t\right)}|\beta_{kpq}^{\left(t-1\right)})$.

To induce sparsity, we place spike‐and‐slab type priors on the elements $\beta_{kpq}^{\left(t\right)}$, which shrink coefficients with weak or uncertain effects toward zero. To enhance mixing and improve parameter identification during estimation, we adopt a continuous shrinkage prior [40] with density

(4) $$\begin{array}{c} p(\beta_{kpq}^{\left(t\right)}|\beta_{kpq}^{\left(t-1\right)})=\pi_{0}(\beta_{kpq}^{\left(t-1\right)})f_{N}(\beta_{kpq}^{\left(t\right)};0,\sigma_{0}^{2})\;+\;\{1-\pi_{0}(\beta_{kpq}^{\left(t-1\right)})\}f_{\text{slab}}(\beta_{kpq}^{\left(t\right)}), \end{array}$$

where $\pi_{0}(\beta_{kpq}^{\left(t-1\right)}):\mathbb{R}\to (0,1)$ is a function of the past parameter. The spike density $f_{𝒩}(\beta_{kpq}^{\left(t\right)};0,\sigma_{0}^{2})$ is assumed to be a Gaussian density centered at zero with small variance $\sigma_{0}^{2}$, ensuring substantial prior mass is placed near zero. In contrast, the slab distribution $f_{\text{slab}}(\beta_{kpq}^{\left(t\right)})$ is specified as a nonlocal distribution with respect to $\beta_{0}=0$.

The nonlocal slab assigns negligible prior mass near zero [36] and concentrates probability away from the origin on both the positive and negative axes. This structure sharpens the distinction between signal and noise while simultaneously separating negative from positive effects. As a result, regression coefficients naturally fall into three interpretable macro‐categories: negative, negligible, and positive. This facilitates interpretation and the identification of population clusters with distinct patterns of covariate influence on risky behaviors. Operatively, we follow Denti et al. [41] and specify $f_{\text{slab}}(\beta_{kpq}^{\left(t\right)})$ as a bimodal mixture of two Gaussians $𝒩(\mu_{-1},\sigma_{-1})$ and $𝒩(\mu_{1},\sigma_{1})$, and suitably weighted by a function that reduces mass near the origin. This induces a three‐component mixture prior

(5) $$\begin{array}{c} p(\beta_{kpq}^{\left(t\right)}|\beta_{kpq}^{\left(t-1\right)})\;=\;\pi_{0}(\beta_{kpq}^{\left(t-1\right)})f_{N}(\beta_{kpq}^{\left(t\right)};0,\sigma_{0}^{2})\;+\sum_{l\in \{-1,1\}}\pi_{l}(\beta_{kpq}^{\left(t-1\right)})g_{l}(\beta_{kpq}^{\left(t\right)};\mu_{l},\sigma_{l},\xi ), \end{array}$$

with $\pi_{l}(\beta_{kpq}^{\left(t-1\right)})\in (0,1)$ for l∈{−1,0,1} and such that $\sum_{l=-1}^{1}\pi_{l}(\beta_{kpq}^{\left(t-1\right)})=1$. The functions $g_{-1}(\beta_{kpq}^{\left(t\right)};\mu_{-1},\sigma_{-1},\xi )$ and $g_{1}(\beta_{kpq}^{\left(t\right)};\mu_{1},\sigma_{1},\xi )$ are kernel functions allocating higher mass of probability on the negative and positive semi‐axes, respectively. The detailed prior specification is reported in the Supporting Information, Appendix B.

To model coefficient dynamics, we link the prior mixture probabilities $\pi_{l}(\beta_{kpq}^{\left(t-1\right)})$ at time t>1 to the coefficient value $\beta_{kpq}^{\left(t-1\right)}$ observed at time t−1. We specify an increased probability that each coefficient at time t remains in the same mode as at time t−1, inducing smooth temporal dynamics and avoiding abrupt changes, as the dynamic spike‐and‐slab processes [42]. The precise specification of the mixture probabilities and their evolution is provided in Appendix B of the Supporting Information, together with illustrative examples of the resulting prior coefficient densities (Figure S2). For lower‐dimensional parameterized models, Gaussian random walk priors on $\beta_{kpq}^{\left(t\right)}$ are also an option worth being considered.

## 3 Online Bayesian Inference via SMC

### 3.1 Modular Framework for Sequential Inference

Given the sequential nature of the health surveillance systems' data collection, our goal is to infer the joint distribution of group indicators and cluster‐specific regression parameters as new data become available. We then first introduce key notation and emphasize the modular structure of the model. We then develop a forward inferential strategy to obtain our target inferences efficiently.

For a time of interest T, we denote with $B^{\left(T\right)}$ the set of group coefficient matrices $B^{\left(T\right)}=\{B_{1}^{\left(T\right)},\ldots ,B_{K}^{\left(T\right)}\}$ and with $s^{\left(T\right)}$ the membership indicators $s^{\left(T\right)}=(s_{1}^{\left(T\right)},\ldots ,s_{N_{T}}^{\left(T\right)})$. For all the elements considered, the superscript (1:T) will indicate all time points t∈{1,…,T}. For instance, ${𝒟}^{\left(1:T\right)}$ will refer to all the observed data up to time T, that is, ${𝒟}^{\left(1:T\right)}=\{X^{\left(1:T\right)},V^{\left(1:T\right)},Y^{\left(1:T\right)},W^{\left(1:T\right)}\}$. We remark that, in our study, multiple observations are gathered at each time point T. Specifically, ${𝒟}^{\left(T\right)}=\{X^{\left(T\right)},V^{\left(T\right)},Y^{\left(T\right)},W^{\left(T\right)}\}$ denotes the collection of data corresponding to the $N_{T}$ respondents whose information becomes available at time T. In the case of the PASSI survey, where data are collected on a monthly basis, $N_{t}$ is typically on the order of several thousand observations.

Our goal is to recover the *filtering* distribution $p(s^{\left(T\right)},B^{\left(T\right)}|{𝒟}^{\left(1:T\right)})$, for any T. As is common in Bayesian inference, we approximate the target distribution via a Monte Carlo method. Specifically, we design an algorithm capable of sampling a sufficiently large number of particles from $p(s^{\left(1:T\right)},B^{\left(1:T\right)}|{𝒟}^{\left(1:T\right)})$, and then numerically integrate out the non‐relevant parameters $s^{\left(1:T-1\right)}$ and $B^{\left(1:T-1\right)}$.

Conditionally on the covariates $X^{\left(1:T\right)}$, $V^{\left(1:T\right)}$, and on the group memberships $s^{\left(1:T\right)}$, the model described in Section 2 assumes two independent data‐generating processes: one for the levels of risky behaviors, $Y^{\left(1:T\right)}$, and another for the occupational text descriptions, $W^{\left(1:T\right)}$. This assumption leads to the following decomposition

(6) $$\begin{array}{ll} p(s^{\left(1:T\right)},B^{\left(1:T\right)}|D^{\left(1:T\right)}) & =p(Y^{\left(1:T\right)}|B^{\left(1:T\right)},s^{\left(1:T\right)},X^{\left(1:T\right)})\\ & \;\times p{\left(Y^{\left(1:T\right)},W^{\left(1:T\right)}\right)}^{-1}\;p(B^{\left(1:T\right)})\\ & \;\times p(s^{\left(1:T\right)}|V^{\left(1:T\right)},W^{\left(1:T\right)}). \end{array}$$

Building on this factorization, estimation proceeds in two stages. In the first stage, we estimate the posterior distribution of group membership probabilities, given the textual and socioeconomic data, that is,

$$\begin{array}{cc} p(s^{\left(1:T\right)}|W^{\left(1:T\right)},V^{\left(1:T\right)}) & \propto \int_{\theta }p(W^{\left(1:t\right)}|\theta^{\left(1:T\right)})\;p(s^{\left(1:T\right)}|\theta^{\left(1:T\right)})\\ & \;\times p(\theta^{\left(1:T\right)}|V^{\left(1:T\right)})\;d\theta . \end{array}$$

In the second stage, inference is performed on the regression coefficients $B^{\left(1:T\right)}$, while updating the latent group assignments $s^{\left(1:T\right)}$ using the activity data $Y^{\left(1:T\right)}$. The distribution $p(s^{\left(1:T\right)}|W^{\left(1:T\right)},V^{\left(1:T\right)})$ estimated in the first stage serves as an informative prior for the mixture weights in the multivariate ordinal regression model $p(Y^{\left(1:T\right)}|B^{\left(1:T\right)},s^{\left(1:T\right)},X^{\left(1:T\right)})$.

Efficient algorithms for estimating the STM provide accurate approximations of $p(s^{\left(1:T\right)}|W^{\left(1:T\right)},V^{\left(1:T\right)})$. We employ computationally efficient logistic‐normal approximations [35] to the posterior topic proportions $p(\theta^{\left(1:T\right)}|W^{\left(1:T\right)},V^{\left(1:T\right)})$, which make sampling from $p(s^{\left(1:T\right)}|W^{\left(1:T\right)},V^{\left(1:T\right)})$ straightforward. This sampling depends on the hyperparameters Γ, Λ, and Ψ, whose specification is discussed in detail in Section 3.2. Finally, the joint posterior distribution $p(s^{\left(1:T\right)},B^{\left(1:T\right)}|{𝒟}^{\left(1:T\right)})$ can be sequentially updated over time, as described in Section 3.3. Posterior samples can be obtained through Hamiltonian Monte Carlo (HMC, e.g., via Stan [43]), Gibbs sampling [44], or SMC methods [45]. In Section 3.4, we detail the custom SMC procedure developed for this study, which achieves substantial computational efficiency compared to alternative approaches, as shown in the simulation results in Section 4.

### 3.2 Specification of STM Hyperparameters

The distribution $p(s^{\left(1:T\right)}|W^{\left(1:T\right)},V^{\left(1:T\right)})$ depends on the hyperparameters Γ, Λ, and Ψ. In particular, the prior distribution $p(\theta^{\left(1:T\right)}|V^{\left(1:T\right)})$ is governed by the hyperparameter matrices Λ and Ψ, while the likelihood term $p(W^{\left(1:T\right)}|\theta^{\left(1:T\right)})$ is determined by the hyperparameter matrix Γ. It is useful to recall that Λ encodes prior expectations about the contribution of covariate effects, that is, socioeconomic indicators, in explaining the occupational group structure. Conversely, the columns $\gamma_{k}$ of Γ define the vocabulary *rules* that characterize each topic, governing the probability of word usage across topics of interest. In view of this, when an external corpus related to the occupational domain is available, Γ can be set by maximizing the likelihood of that corpus, thereby encoding prior vocabulary structure before analyzing the survey texts. Similarly, if auxiliary data on socioeconomic indicators in occupations exist, they may be used to specify the values of Λ, reflecting prior knowledge about occupational groupings.

In practice, when a sufficiently large period of survey responses is available, these hyperparameters can be directly estimated from the data jointly with the logistic‐normal approximations of $p(\theta^{\left(1:T\right)}|W^{\left(1:T\right)},V^{\left(1:T\right)})$. Specifically, we use the textual data W and socioeconomic indicators V observed over an initial training period $1:t^{⋆}$, for a certain $t^{⋆}$ sufficiently large, and apply the non‐conjugate variational expectation–maximization algorithm [35], obtaining regularized point estimates ${\hat{\Gamma }}_{t^{⋆}}$, ${\hat{\Lambda }}_{t^{⋆}}$, and ${\hat{\Psi }}_{t^{⋆}}$ for the hyperparameters. We keep the hyperparameters fixed for all subsequent analyzes, ensuring consistent topic interpretation over time. If the analysis horizon T extends far beyond $t^{⋆}$, such that occupational terminology, the relationships between jobs and socioeconomic, or educational factors may have evolved, it is advisable to re‐estimate ${\hat{\Gamma }}_{T}$, ${\hat{\Lambda }}_{T}$, and ${\hat{\Psi }}_{T}$ using the most recent corpus. This ensures alignment of our prior knowledge with contemporary language use and the evolving socioeconomic structure of occupations.

### 3.3 Online Learning Between Months

We now formulate the two‐stage approach, previously introduced, in a sequential manner to update the estimates for each new time point. Considering the Markovian dynamics of the regression coefficients, as specified in Section 2.3, and the conditional independence of subjects within the latent Gaussian vectors $z_{n}$ and $\theta_{n}$ in Equations (1) and (2), we can decompose the terms in the numerator of Equation (6) across time. Specifically, given fixed hyperparameters, we have

$$\begin{array}{ll} p(s^{\left(1:T\right)},B^{\left(1:T\right)}|D^{\left(1:T\right)}) & \propto \prod_{t=1}^{T}\;\{p(Y^{\left(t\right)}|B^{\left(t\right)},s^{\left(t\right)},X^{\left(t\right)})\;p(B^{\left(t\right)}|B^{\left(t-1\right)})\\ & \;\times L(W^{\left(t\right)},V^{\left(t\right)},s^{\left(t\right)}), \end{array}$$

with $ℒ(W^{\left(t\right)},V^{\left(t\right)},s^{\left(t\right)})=\int_{\theta }p(W^{\left(t\right)}|\theta^{\left(t\right)})p(s^{\left(t\right)}|\theta^{\left(t\right)})p(\theta^{\left(t\right)}|V^{\left(t\right)})\;d\theta$ and $p(B^{\left(1\right)}|B^{\left(0\right)})=p(B^{\left(1\right)})$. We can obtain $p(s^{\left(T\right)},B^{\left(T\right)}|{𝒟}^{\left(1:T\right)})$ by simply noting that

$$\begin{array}{ll} p(s^{\left(T\right)},B^{\left(T\right)}|D^{\left(1:T\right)}) & \propto p(Y^{\left(T\right)}|B^{\left(T\right)},s^{\left(T\right)},X^{\left(T\right)})\;p(B^{\left(T\right)}|B^{\left(T-1\right)})\\ & \;\times p(s^{\left(T-1\right)},B^{\left(T-1\right)}|D^{\left(1:T-1\right)})L(W^{\left(T\right)},V^{\left(T\right)},s^{\left(T\right)}). \end{array}$$

ALGORITHM 1 **Between months filtering algorithm.**

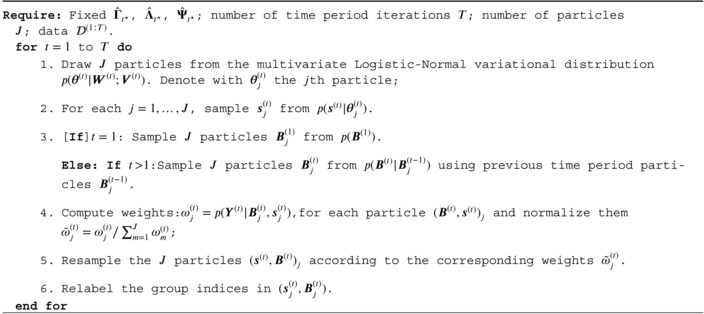

If a sample from $p(B^{\left(T\right)}|B^{\left(T-1\right)})p(s^{\left(T-1\right)},B^{\left(T-1\right)}|{𝒟}^{\left(1:T-1\right)})$ is available in the form of particles; it suffices to sample from $ℒ(W^{\left(T\right)},V^{\left(T\right)},s^{\left(T\right)})$ and re‐sample the pair $\left(B^{\left(T\right)},s^{\left(T\right)}\right)$ with weight $\omega^{\left(t\right)}=p(Y^{\left(T\right)}|B^{\left(T\right)},s^{\left(T\right)},X^{\left(T\right)})$ to obtain a Monte Carlo approximation of the *filtering distribution* $p(s^{\left(T\right)},B^{\left(T\right)}|{𝒟}^{\left(1:T\right)})$, which can then be used for the next time period. The procedure is summarized in Algorithm 1, which takes as input ${\hat{\Gamma }}_{t^{⋆}}$, ${\hat{\Lambda }}_{t^{⋆}}$, and ${\hat{\Psi }}_{t^{⋆}}$, the number of time periods T, and the number of particles J.

The Steps 4 and 5 in Algorithm 1 require updating a multidimensional parameter that also depends on the number of units observed in each time period. As widely recognized in the literature [45, 46], updating static multidimensional parameters often leads to particle degeneracy, resulting in poor samples characterized by a limited number of effective particles. To mitigate this issue, we introduce a set of adjustments, which are detailed in Section 3.4.

To maintain consistency in group membership labels across particles, in Step 6 of Algorithm 1 we apply the relabeling algorithm of Stephens [47] on $s^{\left(t\right)}$, as implemented in the R package mcclust [48]. Based on the resulting relabeling, we consistently align the group indices k=1,…,K in each particle of $B^{\left(t\right)}$.

### 3.4 Within‐Month Update and Parallel Computing

Let us consider a specific iteration of Algorithm 1 corresponding to time period t, focusing particularly on Steps 4 and 5. Since $s^{\left(t\right)}$ is an $N_{t}$‐dimensional vector and $B^{\left(t\right)}$ is a K×P×Q‐dimensional array, the parameter space becomes high‐dimensional when K, Q, or even just the number of observations $N_{t}$ within the period is large. As is well known in the literature, the *curse of dimensionality* causes particles to be widely dispersed, leading to only a handful of normalized weights ${\tilde{\omega }}_{j}^{\left(t\right)}$ with significantly higher values than the others. This results in the resampling of a small number of particles in Step 5 of Algorithm 1, making inference on $\left(s^{\left(t\right)},B^{\left(t\right)}\right)$ infeasible. To address this issue, we replace Steps 4 and 5 with a filtering procedure that sequentially updates the parameter $\left(s^{\left(t\right)},B^{\left(t\right)}\right)$ by incorporating one observation at a time within the month. To mitigate particle degeneracy in the marginal posterior of $B^{\left(t\right)}$, we use a *rejuvenation step* in the algorithm, as is typically done in SMC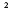 algorithms [49]. The full procedure is detailed in Algorithm 2; herewe focus on the main ideas.

ALGORITHM 2 **Within‐month particle filtering algorithm with rejuvenation.**

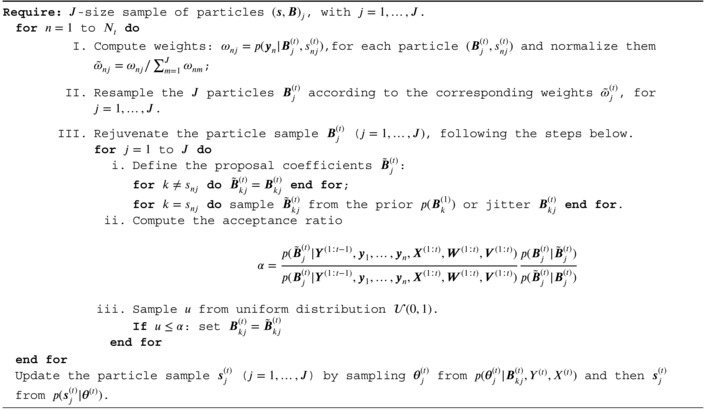

Assume that a sample of size J of $\left(s^{\left(t\right)},B^{\left(t\right)}\right)$ is available from the prior distribution $p(s^{\left(t\right)}|W^{\left(t\right)},V^{\left(t\right)})p(B^{\left(t\right)}|B^{\left(t-1\right)})$. For $n=1,\ldots ,N_{t}$, observations $\left(y_{n}^{\left(t\right)},x_{n}^{\left(t\right)}\right)$ are processed sequentially to update posterior beliefs on group memberships $s^{\left(t\right)}$ and regression coefficients $B^{\left(t\right)}$.

In Steps I–II of Algorithm 2, particle weights are computed from the likelihood contribution of the current observation and used to resample the particle set. While this procedure yields a valid filtering distribution, the finite particle sample can lead to a rapid depletion of diversity in $B^{\left(t\right)}$ as new observations are incorporated, particularly when the number of groups K is large. This phenomenon is known and commonly discussed in SMC literature[45, 50].

We address particle depletion by incorporating a Metropolis‐Hastings rejuvenation step [49] after each filtered observation. This step allows particles to explore the local posterior distribution of $B^{\left(t\right)}$ and helps restore diversity in the particle population. The acceptance ratio α used in Step III ii of Algorithm 2 is described in Appendix C of the Supporting Information.

New proposal values ${\tilde{B}}_{j}^{\left(t\right)}$ are generated either from the prior distribution or by jittering the current particle values. The prior density $p(B_{j}^{\left(t\right)}|{𝒟}^{\left(1:t-1\right)})$ is evaluated at the current state $B_{j}^{\left(t\right)}$ using results from the previous iteration n−1, whereas its value at the proposal ${\tilde{B}}_{j}^{\left(t\right)}$ is computed via numerical integration. Computational details on the evaluation of the prior density and on the acceptance ratio used in Step III ii of Algorithm 2 are provided in Appendix C of the Supporting Information.

The algorithm admits partial parallelization. One strategy may be to execute Step III in parallel for j=1,…,J. However, this strategy requires calculating Steps I–II for all particles, which should number in the thousands to ensure adequate approximation performance. To reduce computational demand while enhancing parameter space exploration, we propose an alternative strategy in which we run multiple independent parallel instances of Algorithm 2, each using a small to moderate number of particles J. Within each instance, the order of observations in ${𝒟}^{\left(t\right)}$ is randomly permuted, leveraging the order‐invariant model specification within each time period t. This strategy improves mixing and reduces sensitivity to local modes while maintaining computational efficiency. To promote effective exploration of the parameter space at the first time period, we approximate the filtering distribution $p(s^{\left(1\right)},B^{\left(1\right)}|{𝒟}^{\left(1\right)})$ using HMC implemented in Stan.

At each time period t, one could instead rely on MCMC‐based posterior approximation schemes, such as those proposed in Goudie et al. [51]. While these methods can improve exploration of the parameter space, they typically require running MCMC until convergence at each time period, which substantially increases computational cost. Our proposal can therefore be viewed as a more computationally efficient alternative, as demonstrated by the simulation studies in Section 4.

## 4 Algorithm Assessment via Simulation Studies

We evaluate the performance of the proposed SMC algorithm by comparing it with several alternatives. Specifically, we examine its ability to approximate the posterior predictive distribution $p({\tilde{y}}_{n}|x_{n},{𝒟}^{\left(1:t\right)})$ for a new subject, given the availability of a sample from the logistic‐normal distribution $p(\theta^{\left(1:t\right)}|W^{\left(1:t\right)},V^{\left(1:t\right)})$.

We mimic the data‐generating process of the motivating application described earlier. For two consecutive periods (t∈{1,2}), we consider a P=4‐dimensional ordinal response variable, similar in structure to the four SNAP variables of the application. We design eight scenarios by varying $\left(N_{1},N_{2})\in \{(200,250),(1000,1200)\right\}$, (Q,K)∈{(3,5),(5,10)}, and a sparsity index ς∈{0.2,0.4} used to control the amount of regression coefficients set to zero. In each scenario, we first randomly set the binary covariate matrix X, the regression coefficients ${\bar{B}}^{\left(1\right)}$ and ${\bar{B}}^{\left(2\right)}$, and the true clustering belonging $\bar{s}$. Then, we replicate for R=20 times the generation of response variables $Y^{\left(1\right)}$ and $Y^{\left(2\right)}$ according to the ordinal multivariate mixture model in Equation (1). All simulations were run in parallel on a Windows machine equipped with an AMD EPYC 7413 40‐Core Processor (2.65 GHz), using 30 cores. Full details of simulation settings are provided in Appendix D of the Supporting Information.

For each replication, different estimation strategies are adopted to approximate the posterior predictive distribution $p(y_{n}|x_{n},{𝒟}^{\left(1:2\right)})$, for the subjects observed in the second period, that is, $n=N_{1},\ldots ,N$, with $N=N_{1}+N_{2}$, conditional on the full observed dataset $\left(X^{\left(1:2\right)},Y^{\left(1:2\right)}\right)$ and given the availability of a sample from a logistic‐normal distribution $p(\theta^{\left(1:2\right)}|W^{\left(1:2\right)},V^{\left(1:2\right)}).$ For all competing algorithms, the logistic‐normal distribution hyperparameters used to sample the *true* $\theta_{n}$, for n=1,…,N, are available. The topic model results are provided as a prior distribution for clustering, that is, the comparisons refer to the ability to correctly update the prior belief on $B^{\left(2\right)}$, and s after observing $X^{\left(1:2\right)}$ and $Y^{\left(1:2\right)}$.

As a gold‐standard benchmark, we consider the predictive distribution sample obtained from HMC run in Stan [43]. We denote this benchmark as $p_{\text{HMCF}\mathrm{ULL}}(y_{n}|x_{n},{𝒟}^{\left(1:2\right)})$. This method does not allow for online updates and requires the full dataset from the entire period observed to estimate jointly $B^{\left(1\right)}$, $B^{\left(2\right)}$, and $\theta_{n}$ for all n=1,…,N. This strategy offers good sampling qualities, but it could be time and memory‐consuming, especially in higher dimensions (may be them Q, T, $N_{t}$, or K). In the simulations, the hmcfull is run using 30 parallel chains with 2000 iterations each, discarding the first 1850 iterations as warm‐up, leading to a sample with 4500.

We use $p_{\text{HMCF}\mathrm{ULL}}(y_{n}|x_{n},{𝒟}^{\left(1:2\right)})$ to assess the quality of approximation of alternative algorithms. As evaluation metric, we consider the empirical Kullback–Leibler divergence between $p_{\text{HMCF}\mathrm{ULL}}(y_{n}|x_{n},{𝒟}^{\left(1:2\right)})$ and the predictive distributions of alternative algorithms, averaged across all subjects in period t=2. We rely on the implementation available in the R package kldest [52], with divergence close to zero indicating that the method closely approximates the full HMC predictive distribution.

As a first approach, we consider the predictive distribution approximated by our proposed method, described in Algorithms 1 and 2, which generates J=4500 particles to approximate the posterior predictive distribution at t=2. Following the recommendations in Section 3.4, we employ parallel computing by running 30 parallel instances with 150 particles each. The resulting distribution is denoted as $p_{\text{SMC}}(y_{n}|x_{n},{𝒟}^{\left(1:2\right)})$. We compare our results against two types of competitors: (i) Laplace approximation methods using the full sample, which are known for their computational efficiency, and (ii) sequential sampling‐based methods, which typically offer higher approximation accuracy. The Laplace approximation based on the full periods is denoted by $p_{\text{LF}\mathrm{ULL}}(y_{n}|xn,{𝒟}^{\left(1:2\right)})$. We obtain this distribution using the cmdstanr implementation of the Laplace approximation algorithm, applied to the same model and data used for $p_{\text{HMCF}\mathrm{ULL}}(y_{n}|x_{n},{𝒟}^{\left(1:2\right)})$. This implementation jointly estimates the parameters $B^{\left(1\right)}$, $B^{\left(2\right)}$, and $\theta_{n}$ for all $n=N_{1},\ldots ,N$, used to get 4500 draws from the respective posterior predictive distribution. The remaining two competitors enable sequential inference through a two‐step procedure. In the first step, we fit a flexible mixture model to the posterior samples of $B^{\left(1\right)}$, obtaining a closed‐form approximation of $p(B^{\left(1\right)}|{𝒟}^{\left(1\right)})$. This approximation, combined with the dynamic specification in Equation (5), provides an approximate prior for $B^{\left(2\right)}$ conditional on $B^{\left(2\right)}$ conditional on first period data ${𝒟}^{\left(1\right)}$. In the second step, we estimate the predictive distribution at t=2 using either a Stan implementation of HMC or the Laplace approximation algorithm, obtaining 4500 draws from *p*_hmc2step_ $\left(y_{n}|xn,{𝒟}^{\left(1:2\right)}\right)$ and *p*_l2step_ $\left(y_{n}|x_{n},{𝒟}^{\left(1:2\right)}\right)$, respectively, for $n=N_{1},\ldots ,N$. These two approaches represent computationally efficient alternatives to the full‐sample algorithms, as they require an approximation of $p(B^{\left(t\right)}|{𝒟}^{\left(1:(t-1)\right)})$ as input, while updating only the parameters at time t (in this case, t=2) like our proposed method.

Simulation results assessing the quality of the predictive approximations are reported in the left panel of Figure 3. As expected, Laplace‐based methods exhibit the largest discrepancies, likely due to their limited ability to capture mixture structures. In contrast, the SMC and HMC two‐step methods provide comparable approximation quality, with our proposed approach showing slightly better performance. This advantage appears more systematic as the number of nonzero parameters increases, that is, in scenarios with larger (K,Q) values or smaller ς.

**FIGURE 3:**
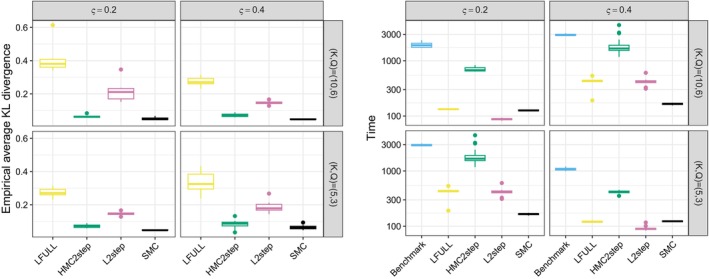
Boxplots of the distribution over R=20 simulation replications of the average KL divergence between the benchmark predictive posterior samples provided by hmcfull and those generated by the competing models (left), and of the time (in seconds with axis in base‐10 logarithmic scale) required to generate predictive posterior samples for subjects at t=2 for each competing model (right), evaluated across varying ς, K, and Q.

Other than providing better quality in approximation, the proposed method also offers a significant advantage in terms of computational efficiency. The right panel of Figure 3 shows the time (in seconds) required by each algorithm to generate predictive samples for $n=N_{1},\ldots ,N$. The time required to estimate $B^{\left(1\right)}$ is omitted whenever possible; however, for the non‐sequential algorithms (hmcfull and lfull) this step cannot be isolated, so the reported times include the joint estimation of $B^{\left(1\right)}$ and $B^{\left(2\right)}$. This does not significantly disadvantage the full‐sample methods, given the limited number of periods (T=2). In fact, the advantage of sequential approaches emerges as T increases: They update previously estimated quantities using new data, leading to a computational cost that grows roughly linearly in T, whereas full‐sample methods must reprocess all past data, causing their burden to escalate quickly. Our SMC approach demonstrates substantial computational savings relative to Hamiltonian Monte Carlo methods, primarily because it avoids the costly warm‐up phases. Furthermore, its fully parallelizable implementation enables performance comparable to faster, though less accurate, alternatives such as the Laplace approximations. Notably, this remains true even in highly parameterized settings, where the SMC algorithm continues to perform competitively in both speed and accuracy.

Additional simulation scenarios, yielding consistent conclusions across settings with smaller N, are reported in Figures S3, S4 in Appendix D of the Supporting Information.

## 5 Health Risk Behaviors in Italy

### 5.1 Health Surveillance System Data

We analyze data from the Italian health surveillance system PASSI. The population of reference is Italian adults aged 18–69. Sampling is stratified by sex and age within each Local Health Unit, using population health registries, with at least six sex‐by‐age strata (i.e., men aged 18–34, 35–49, and 50–69, and women aged 18–34, 35–49, and 50–69). This design supports representativeness and reliable estimates at the LHU‐sex‐age level. Except for Lombardy, all Italian regions participate. Each year, PASSI involves more than 90% of LHUs, covers over 90% of the resident population, and achieves response rates above 85%, with refusal rates below 10% [53]. Because PASSI is a repeated cross‐sectional surveillance system and the anonymized data do not allow respondent tracking across survey waves, repeated observations from the same individual could not be explicitly modeled, although such occurrences are expected to be rare. For additional information, please refer to Baldissera et al. [13] and the website of the Istituto Superiore di Sanità [53]. We analyze data from 2014 to 2023, as job‐related information has been available in the survey since 2014. Approximately 35% of the observations correspond to unemployed respondents, who are excluded from the analysis, yielding a final sample of 133,485 observations. The variables used in the model of Figure 2, that is, the demographic covariates $x_{n}$, socioeconomic characteristics $v_{n}$, and behavioral outcomes $y_{n}$, are defined in Table 1. Thresholds $\tau_{pc}$ were set according to the number of categories of each ordinal outcome, so that the corresponding cumulative probabilities of a standard normal distribution are approximately balanced between adjacent thresholds. Specifically, we used $\left(\tau_{p1},\tau_{p2},\tau_{p3},\tau_{p4},\tau_{p5})=(-1,-0.5,0,0.5,1\right)$ for smoking, $\left(\tau_{p1},\tau_{p2},\tau_{p3})=(-0.75,0,0.75\right)$ for nutrition and alcohol consumption, and $\left(\tau_{p1},\tau_{p2})=(-0.5,0.5\right)$ for physical inactivity. The classification of smoking and alcohol consumption followed the guidelines of the Istituto Superiore di Sanitã (Italian National Institute of Health) [53]. In contrast, the indicators for nutrition and physical activity were retained as originally recorded in the questionnaire.

**TABLE 1:** Description of the variables included in the analysis.

	Variable	Description
$x_{n}$	Sex	Male; female
	Age	[18,30); [30, 50); [50, 69]
	Macro area	Center; islands; north‐east; north‐west; South
$v_{n}$	Educational level	Low: if below high school; high: otherwise
	Economic problem	0: if the respondent easily meets financial needs; 1: otherwise
$y_{n}$	Smoke	0: never smoked, 1: currently smokes less than a cigarette, 2: currently smokes 1–4 cigarettes, 3: currently smokes 5–9 cigarettes, 4: currently smokes 10–19 cigarettes, 5: currently smokes at least 20 cigarettes (per day)
	Nutrition	3: No fruit; 2: 1–2 fruit portions; 1: 3–4 fruit portions; 0: 5+ fruit portions (per day)
	Alcohol	Sum of three binary indicators: high alcohol consumption (more than two drinks per day for men and more than one drink per day for women), drinking outside meals, and at least one episode of binge drinking, where a value of 1 indicates the presence of risky behavior. The variable takes values in the set {0,1,2,3}.
	Physical activity	0: Intense; 1: moderate; 2: no physical activity (in the last 30 days).

For the textual data about occupation, we examine three variables related to the respondents' employment. The first stems from the query: “Can you tell me what you do for a living?”, that is, an open‐ended question. The second involves the classification of the declared job according to ISTAT coding (Italian National Institute of Statistics) [28], while the third one delineates nineteen occupational sectors defined both in Tables S1, S2 of Appendix A in the Supporting Information. While the latter two provide structured categorical information, they may fail to capture the rich contextual and semantic nuances contained in the open‐ended responses. For each respondent n, we therefore integrate all three occupational sources into a unified textual description of the job. After removing stopwords, we construct a document‐term matrix containing all unigrams appearing in these fields. Because many occupations are repeated, we retain only the unique combinations of texts and $v_{n}$ for the estimation of the STM, ensuring that two respondents with identical occupational descriptions $w_{n}$ and socioeconomic characteristics $v_{n}$ share the same distribution $\mathrm{Pr}(\theta_{n}|W,V)$. This does not preclude assigning different group proportions to these respondents after observing the behavioral data Y.

Following Roberts et al. [35], we selected the STM topic number by fitting models with K=15,…,35 and comparing their held‐out likelihood and semantic coherence. The model with K=30 topics was chosen for its strong interpretability‐performance trade‐off and as part of a deliberate overparameterization strategy. Using a relatively large number of topics helps capture fine‐grained occupational and socioeconomic nuances, yielding a flexible representation of occupational heterogeneity. At the same time, the shrinking non‐local prior regularizes the mixture model by automatically down‐weighting redundant or weakly identified topics. This shrinkage is particularly relevant because, despite the large overall sample size, some occupation‐by‐time combinations may contain few observations. Rather than estimating separate effects for isolated occupation‐month cells, the model stabilizes inference by borrowing strength across latent occupational groups and adjacent time periods. In this way, the model captures genuine variation while merging topics with similar behavioral patterns, producing a parsimonious yet nuanced view of occupational groups. We train the STM using data collected up to December 2019, corresponding to $t^{⋆}=72$, keeping fixed ${\hat{\Gamma }}_{t^{⋆}}$, ${\hat{\Lambda }}_{t^{⋆}}$, and ${\hat{\Psi }}_{t^{⋆}}$ for all t=1,…,120. Further details on prior specification and computational aspects are provided in Appendix E of the Supporting Information.

### 5.2 Predictive Assessment of Occupational Information

To assess the predictive contribution of the free‐text job description, we compare our proposed specification against three benchmarks using the first two periods of available data. We split the observations into a training set (70%) and a test set (30%), fit each model on the training set, and evaluate predictive performance on the held‐out test set using the posterior predictive log‐likelihood.

The first competing model is an *Uninformed Mixture*. In this benchmark, the regression model highlighted by the left red dashed square in Figure 2 remains unchanged, but the prior for the mixture weights $\theta_{n}$ is not informed by occupation‐related variables. Specifically, we impose a symmetric Dirichlet prior, which is standard in mixture modeling [22]. This specification allows for latent group heterogeneity, assessing whether accounting for such heterogeneity alone is sufficient to replicate the predictive gains of our proposed model. To guarantee a fair comparison, we set the number of latent groups to K=30, matching our proposal.

The second competing model is a *Standardized‐Variables‐Informed Mixture*. In this specification, the Dirichlet prior on the mixture weights $\theta_{n}$ for each respondent n is centered on the class identified by combining that respondent's occupational sector and ISTAT job classification. Thus, unlike our proposed model, this alternative informs the mixture weights using only the two standardized occupational variables, ignoring potential similarities in the textual job descriptions. A fully crossed specification based on these variables would involve up to 9×19 combinations, many of which would be sparsely populated and computationally burdensome to estimate. Therefore, we construct the classes using the 29 most frequent sector‐ISTAT combinations and add a 30th residual class for all remaining combinations. As an additional baseline, we also report the performance of the prior predictive distribution. This benchmark evaluates predictions before updating the model with the observed training data, thereby providing a reference point for the predictive gains achieved through posterior learning.

For each model, we obtain posterior draws from the predictive distribution and compute the predictive log‐likelihood on the held‐out test observations. More specifically, for each held‐out respondent, we calculate the difference in the posterior predictive log‐likelihood relative to the best‐performing model. We then average these individual‐level differences over the entire test set.

As expected, all fitted models improve substantially over the prior predictive distribution. The proposed approach achieves the highest out‐of‐sample performance. Compared to this proposed model, the prior predictive distribution performs significantly worse, showing an average log‐likelihood difference of −2.136 (standard error 0.438). The two alternative mixtures, the *Uninformed Mixture* and the *Standardized‐Variables‐Informed Mixture*, exhibit similar predictive performance, yielding average differences from our proposed approach of −0.4722 (standard error 0.228) and −0.5091 (standard error 0.256), respectively. This similarity suggests that the uninformed mixture is flexible enough to recover predictive patterns comparable to those obtained by simply centering the weights on the most frequent standardized classifications. However, the superior performance of our proposed approach over both benchmarks demonstrates that the free‐text job descriptions provide significant additional predictive information. Unlike the alternative that relies solely on frequency‐based standardized classes, our proposed model allows the mixture weights to adapt through posterior learning while actively exploiting the occupational and contextual similarities embedded in the text.

### 5.3 Results

The algorithms presented in Section 3 allow us to investigate the filtering distribution $p(s^{\left(t\right)},B^{\left(t\right)}|{𝒟}^{\left(1:t\right)})$, for any t=1,…,T of interest. This joint distribution captures how respondents are grouped, through the posterior of $s^{\left(t\right)}$, based on both prior information from occupational descriptions $W^{\left(t\right)}$ and observed behavioral profiles derived from the SNAP observations $Y^{\left(t\right)}$. Jointly examining $s^{\left(t\right)}$ and $B^{\left(t\right)}$ provides insights into how identified groups evolve and differ in their behavioral patterns.

For instance, Figure S5 in the Supporting Information shows the posterior distributions of the coefficients $\beta_{1k3}^{\left(1\right)}$, for k=1,…,K, highlighting the heterogeneity across latent groups in the effect of belonging to the (50,69] age class on smoking‐related risky behavior. At t=1, it is already evident that being an older worker increases the probability of smoking among skilled metal‐industry workers, while the opposite effect is observed for farmers and agricultural workers. Although this representation underscores the flexibility of the mixture structure in capturing heterogeneous effects across groups, it is limited in conveying temporal evolution. Moreover, the dimensionality of the parameters is extremely high (depending on P, Q, K, and $N_{t}$), requiring careful consideration in summary representation and visualization.

As a more interpretable alternative, we focus on the *filtered predictive distribution* for a *profile*, that is, a hypothetical subject with index ñ, unobserved in the sample but characterized by their own job text description and sociodemographic features. Focusing on predictive distributions of profiles rather than latent parameters offers several advantages. First, the predictive distribution is an invariant posterior summary that inherently accounts for the relative weights of joint realizations of $\left(s^{\left(t\right)},B^{\left(t\right)}\right)$. More importantly, it shifts attention to the probability scale of observed responses, allowing a direct and interpretable assessment of the probabilities of risky behaviors and their temporal evolution as new data become available. For a profile, we define the target distribution as

$$\begin{array}{l} p(y_{\tilde{n}}|x_{\tilde{n}},D^{\left(1:t\right)})=\int p(y_{\tilde{n}}|x_{\tilde{n}},s_{\tilde{n}},B^{\left(t\right)})\;dp(s_{\tilde{n}},s^{\left(t\right)},B^{\left(t\right)}|w_{\tilde{n}},v_{\tilde{n}},D^{\left(1:t\right)}) \end{array}$$

where the hypothetical subject ñ may belong to any period of interest t=1,…,T.

In this framework, any individual of interest can, in principle, be monitored. However, to illustrate the key features of the model, it is useful to select profiles that represent specific socioeconomic environments, that is, choosing particular values of $w_{ñ}$ and $v_{ñ}$, that emphasize the latent relationships within the data. We identify such representative profiles using the findThoughts function from the stm package [39], which retrieves the occupational and socioeconomic profiles most strongly associated with a given topic. Although, the socioeconomic environment is selected from observed individuals, our analysis focuses on profiles whose demographic characteristics $x_{ñ}$ are varied to explore how the predictive distribution of responses changes accordingly. Understanding how the influence of demographic covariates varies across socioeconomic contexts is crucial for both sociological interpretation and policy design. Through this framework, our model provides quantitative assessments of how sociodemographic factors shape the predicted probabilities of risky behaviors, together with their associated uncertainty.

To provide an example, we present in Figure 4 the temporal dynamics of the estimated probabilities of risky behaviors for three representative profiles of young male individuals from north‐east Italy, where, according to the ordinal probit specification, probabilities of risky behaviors are evaluated at the cutpoints 0 for smoking and alcohol (i.e., starting to smoke and exhibiting at least one risky alcohol‐related behavior respectively), 0.5 for nutrition (i.e., consuming fewer than five portions of fruit per day), and 0.75 for physical activity (i.e., being sedentary). The width of the credible intervals should also be interpreted as reflecting the amount of information available for each occupational profile and time period. Profiles that are less frequent or less clearly separated from other latent groups tend to display wider posterior uncertainty. Consequently, our conclusions are based on persistent patterns in the posterior predictive distributions rather than on isolated occupation‐month estimates. We can note that before the COVID‐19 pandemic, skilled metal industry workers exhibited higher smoking propensities, which later converged to the levels observed for qualified educators and unskilled agricultural workers, displaying comparable post‐pandemic dynamics. Instead, qualified educators have a higher likelihood of alcohol consumption, while skilled metal industry workers tend to be less physically active.

**FIGURE 4:**
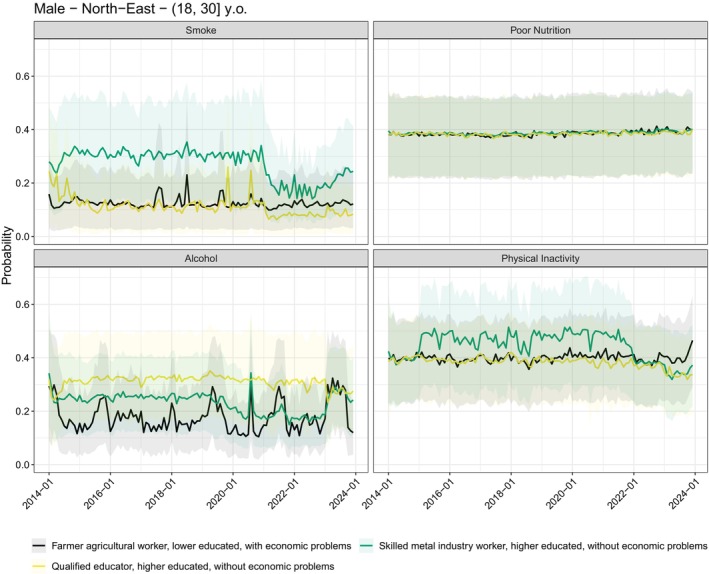
Estimated risk probabilities by year for each SNAP behavior, for 18–30‐year‐old males from north‐east Italy and three latent job profiles. The shaded areas represent the 90% credible intervals.

Clearly, it is just sufficient to consider a specific t to focus on a cross‐sectional perspective. We do this by considering the relative risk, defined here as $p_{gp}^{t}/p_{\cdot p}^{t}$, where $p_{\cdot p}^{t}$ represents the prevalence in risky behavior p of a baseline group. Such relative risks are represented in Figure 5, where prevalences of the baseline categories are computed by considering the strata of [18,30]‐year‐old females living in the center macro area marginal with the same occupation characteristics. The influence of territorial context appears to differ across occupational categories, while other age and sex factors display more stable and unidirectional effects on risky behaviors, with some exceptions. Being from the South emerges as a risk factor for smoking propensity across most hypothetical individuals, particularly among those with higher education levels. Conversely, being male appears to be a protective factor for nearly all occupations, except for those related to industry. These protective effects appear to be more pronounced for occupations characterized by a higher education and better economic conditions.

**FIGURE 5:**
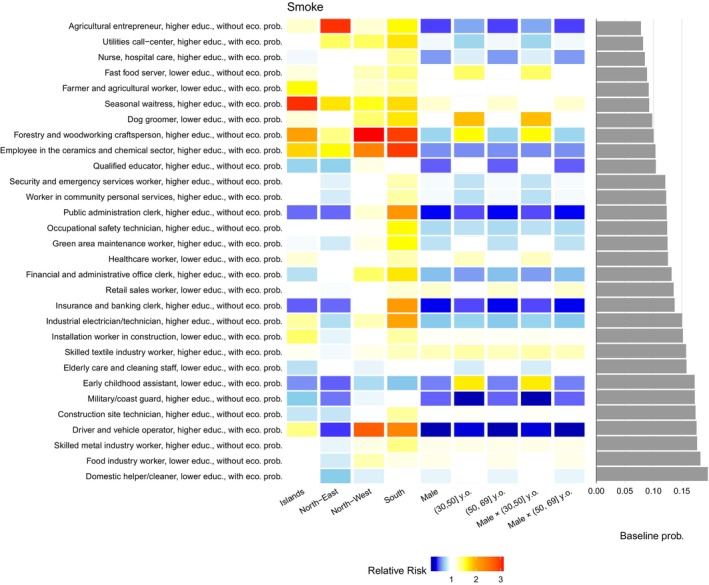
Smoking relative risk for each job group and socio‐demographic covariates for December 2023. Reference individual: [18, 30] female from the center macro area.

The free‐text occupational description then contributes information beyond the standardized occupational sector and ISTAT job classification, which define broad categories and may group individuals with different tasks and work environments. For example, we considered the 2023 annual average posterior predictive probabilities for two respondents sharing the same sector (health care) and ISTAT classification (technical professions), but reporting different free‐text occupations: “nurse” and “physical therapist.” These profiles are assigned to different latent groups, respectively, “Dog groomer, lower educated, with economic problems” and “Healthcare worker, lower educated, with economic problems.” The annual average probability of physical inactivity in 2023 is 0.536 for the nurse profile, and 0.466 for the physical therapist profile, while the corresponding smoking probabilities are 0.162 and 0.104. No relevant differences were found for alcohol consumption or poor nutrition. This example shows that free text can refine occupational grouping even when the standardized variables coincide. In Appendix E of the Supporting Information, the posterior distributions of a coefficient for these two latent groups are shown.

These findings suggest that the relationship between socioeconomic status and SNAP behaviors is not straightforward, as not often recognized in the epidemiological literature [54, 55]. This complexity is, however, well recognized in clinical medicine and in behavioral and medical sociology, where the interplay between individuals and their environments is understood as inherently non‐trivial [56, 57, 58]. Structural conditions, micro‐environmental contexts, and contextual resources can exert both protective and risk‐enhancing effects, shaping health‐related behaviors in ways that are far from simple. Vulnerability does not uniformly translate into unhealthy behaviors, and lifestyle factors, including occupational context, also shape such patterns [59].

The model also permits joint analyzes of behaviors. Figure 6 presents the posterior risk probabilities for alcohol consumption and physical inactivity across demographic covariates. In the South (colored in yellow), physical inactivity is pronounced among unskilled agricultural workers, though it is also evident in other occupational groups. Skilled industry workers exhibit a relatively high propensity for alcohol consumption. Conversely, qualified educators in the north‐east display higher alcohol consumption tendencies, especially among younger individuals, as also shown in Figure S7 in the Supporting Information. From the perspective of risky behaviors, it is clear that hazardous alcohol consumption is more pronounced among younger age groups overall. While this can be partly explained by the different lifestyles people adopt at various stages of life, it is interesting to note that differences also emerge between sexes. The analyzes suggest a tendency toward a higher risk of alcohol consumption among young women compared to men, a pattern particularly pronounced in the north‐eastern regions of the country. In the literature, this association is well documented among adolescents [60, 61], and more recently it has also been observed among young women in Italy, coherently with our study [62], and in the United States [25].

**FIGURE 6:**
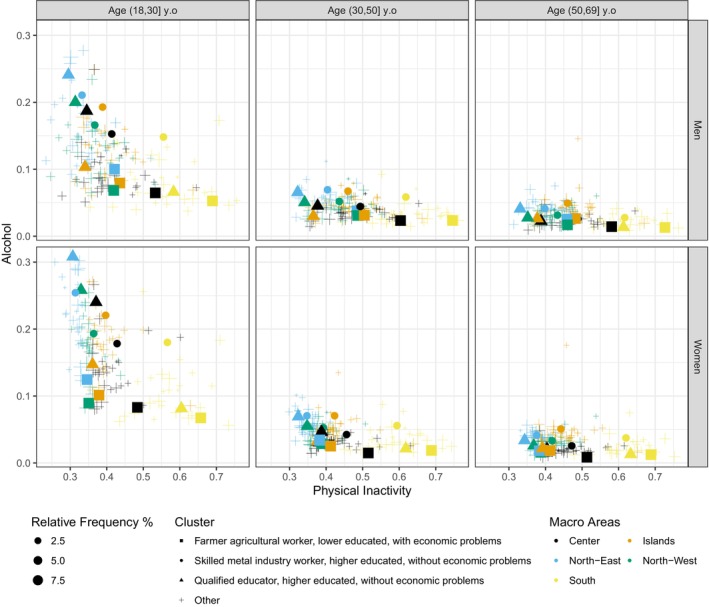
Estimated probabilities of engaging in risky alcohol consumption and physical inactivity, by sex, age group, macro area, and three latent job groups.

These findings highlight the importance of policymakers developing targeted health promotion strategies. Interventions could prioritize increasing physical activity in the southern regions via investments in accessible public spaces and infrastructure, workplace wellness programs, and community‐based initiatives encouraging adult participation in sports or active transport. In the northern regions, efforts should focus on mitigating alcohol‐related risks by implementing awareness campaigns, strengthening preventive education in schools and workplaces, or enforcing stricter regulations on alcohol availability and marketing.

## 6 Conclusion and Future Research Directions

This study proposes a comprehensive statistical framework to examine how occupational factors in combination with socioeconomic conditions influence health‐related risky behaviors in Italy. By integrating structural topic modeling with dynamic multivariate ordinal probit models and a novel Bayesian online learning algorithm, the approach allows for a detailed characterization of heterogeneous and time‐evolving health outcomes in large‐scale data. The complexity of the proposed modeling strategy mirrors the intrinsic complexity of the phenomenon under investigation and aligns with a growing movement in epidemiology that emphasizes the importance of embracing complexity [34, 54, 63]. In the existing literature, analyzes typically focus either on socioeconomic determinants or on occupational characteristics, often treating them as separate domains. In contrast, our approach reveals that such relationships are strongly modulated by occupational conditions and evolve over time, underscoring the importance of modeling contextual and temporal heterogeneity explicitly. It provides individualized analyzes based on representative respondents and can inform ongoing surveillance of behavioral risk factors, allowing analyzes during periods of limited data availability (e.g., the COVID‐19 pandemic) and the identification of trend changes (e.g., before and after the COVID‐19 pandemic). More broadly, the methodology extends naturally to clinical and biomedical settings characterized by multivariate health outcomes, latent heterogeneity, and the availability of unstructured textual information.

Despite its strengths, several limitations remain. Macro‐territoriality has been included within the model through dummy components, ensuring that broad spatial differences are accounted for. However, the surveillance system could also support finer‐grained analyzes at the micro level through spatial models. Achieving this would require further methodological effort in model parameterization and the development of dedicated algorithms for spatio‐temporal inference. The current setup performs filtering rather than full and retrospective smoothing, meaning that continuous updating of estimates is possible as new data arrive, but additional methodological efforts are needed for backward dynamic inference. Although, the model is designed at the individual level, policy interest may also lie in macro and aggregated groups. Since the model operates on individuals, upward aggregation is possible, allowing users or policymakers to dynamically create higher‐level groupings when needed. However, such operations entail substantial computational effort. To make the model's results fully accessible and actionable, a simplified and user‐friendly analytical interface would be required [58]. These developments, together with further optimization of computational routines, are left for future research.

In conclusion, while further extensions are warranted, the proposed approach addresses a major gap in the joint analysis of health behaviors and occupational contexts in Italy. Moreover, the methodology offers a general and novel statistical template applicable to a broad range of health and biomedical research settings, including other countries with comparable behavioral risk‐factor surveillance systems.

## Author Contributions

L.S.: conceptualization, methodology, software, formal analysis, writing – original draft, writing – review and editing, visualization. M.S.: conceptualization, methodology, software, formal analysis, data curation, writing – original draft, writing – review and editing, visualization. A.A.: conceptualization, data curation, writing – review and editing, visualization. S.C.: conceptualization, writing – review and editing, supervision, funding acquisition.

## Funding

Mattia Stival is funded by the European Commission grant 101136652. The five Horizon Europe projects, GO GREEN NEXT, MOSAIC, PLANET4HEALTH, SPRINGS and TULIP form the Planetary Health Cluster. The views and opinions expressed are only those of the authors and do not necessarily reflect those of the European Union or the European Commission. Neither the European Union nor the European Commission can be held responsible for them.

## Conflicts of Interest

The authors declare no conflicts of interest.

## Supporting information



Additional supporting information may be found in the online version of the article at the publisher's website. It includes job‐related covariate tables, prior specification, and computational aspects of the proposed methodology, as well as further simulation results and additional analyses for the Italian PASSI application.

**Appendix A:** Job variables.
**Table S1:** Occupational sector classification labels.
**Table S2:** ISTAT job classification labels.
**Figure S1:** Prevalence ratio between the group‐specific prevalence and the overall population prevalence of exhibiting at least one risky behavior by demographic variable (*x*‐axis) and ISTAT job category (*y*‐axis).
**Appendix B:** Details on spike‐and‐slab dynamic prior.
**Figure S2:** Example of prior density evolution for regression coefficients $\beta_{kpq}^{\left(1\right)}$‐$\beta_{kpq}^{\left(2\right)}$ for two groups k∈{3,8}.
**Appendix C:** Computational details of SMC algorithms.
**Appendix D:** Details on simulation experiments and additional results.
**Figure S3:** Boxplots of the distribution over R=20 simulation replications of the average KL divergence between the benchmark predictive posterior samples provided by HMCFULL and those generated by the competing models, evaluated across varying sparsity levels ζ and parameter dimensions K and Q. The number of subjects is $N_{1}=250$ and $N_{2}=300$.
**Figure S4:** Boxplots of the distribution across R=20 simulation replications of the time (in seconds with axis in base‐10 logarithmic scale) required to generate predictive posterior samples for subjects at t=2 for each competing model, evaluated across varying sparsity levels ζ and parameter dimensions K and Q. The benchmark indicates the log‐time distribution associated with HMCFULL. The number of subjects is $N_{1}=250$ and $N_{2}=300$.
**Appendix E:** Additional application settings and results.
**Table S3:** Description of the representative subjects founded by the findThoughts function from the stm package. The label is made considering the related text and socio‐economic covariates used in the STM.
**Figure S5:** Posterior distributions of the coefficients $\beta_{1k3}^{\left(1\right)}$ (k=1,…,30) associated with the (50, 69] age class and smoking‐related risky behavior.
**Figure S6:** Estimated risk probabilities by year for each SNAP behavior, for a hypothetical 30–50‐year‐old female from south Italy and three latent job groups.
**Figure S7:** Alcohol relative risk for each job group and socio‐demographic covariates for December 2023.
**Figure S8:** Estimated probabilities of engaging in risky smoke use and physical inactivity, by sex, age group, macro area, and three latent job groups.
**Figure S9:** Posterior distributions of the coefficients $\beta_{\mathrm{4k3}}^{(120)}$, for *k* = 1, …, 30, associated with the effect of the (50,69] age class on physical inactivity.

## Data Availability

The data that support the findings of this study are available from Istituto Superiore di Sanità. Restrictions apply to the availability of these data, which were used under license for this study. Data are available from the author(s) with the permission of Istituto Superiore di Sanità.
