# Supplementary material for: Modeling Heterogeneity in Health Risk Behaviors With a Dynamic Mixture Model Informed by Textual Occupational Data
Source: Stat Med. 2026 Aug 10;45(18-19):e70691. doi: 10.1002/sim.70691 (PMC13457518; doi:10.1002/sim.70691)
Supplement: Supplementary file 1 [file SIM-45-0-s001.pdf]

Received: Added at production | Revised: Added at production | Accepted: Added at production
DOI: xxx/xxxx

ARTICLE TYPE

Supporting Information to "Modeling heterogeneity in health risk behaviors with a dynamic mixture model informed by textual occupational data"

Lorenzo Schiavon[1,2] | **Mattia Stival**[3] | **Angela Andreella**[3] | **Stefano Campostrini**[3]

[1]Department of Statistical Sciences, University of Padova, Padova, Italy
[2]Department of Mathematics, University of Padova, Padova, Italy
[3]Department of Economics, Ca' Foscari University of Venice, Venice, Italy

Correspondence
Corresponding author Lorenzo Schiavon, Email: lorenzo.schiavon@unipd.it

Present address
Department of Statistical Sciences, Via C. Battisti, 241, 35121 Padova, Italy

Abstract

Appendix A includes job-related covariate information. Appendix B reports details on spike-and-slab dynamic prior specified on regression coefficients. In Appendix C computational aspects of the SMC algorithm are reported. Appendix D provides additional details and results of the simulation experiments. Appendix E reports computational and prior settings and additional results related to the Italian PASSI data application.

Appendix A | JOB VARIABLES

Table 1 shows the classification of occupational sectors, while Table 2 refers to the job classification according to the ISTAT (https://professioni.istat.it/sistemainformativoprofessioni/cp2011/).

Figure 1 illustrates how the observed ratio between the prevalence of individuals with at least one risky behavior within a given group and the corresponding prevalence in the total population varies across demographic groups and job categories, the latter defined by ISTAT (Italian National Institute of Statistics [1]), i.e., Table 2. Distinct patterns emerge: intellectual workers tend to adopt healthier lifestyles, whereas manual laborers show a higher prevalence of risky behaviors, especially in southern regions.

 © Copyright Holder Name

Table S1 Occupational sector classification labels

Value	Label
1	Agriculture
2	Food industry
3	Metalworking industry
4	Electrical-electronic industry
5	Textile and clothing industry
6	Chemical and ceramic industry
7	Wood and paper industry
8	Other manufacturing industries
9	Construction
10	Energy, gas, water, telecommunications
11	Trade and public services
12	Transport
13	Banking and insurance
14	School and university
15	Health care
16	Public administration
17	Business services
18	Personal services
19	Law enforcement/military

Table S2 ISTAT job classification labels

Value	Label
1	Entrepreneurs, senior executives, and legislators
2	Intellectual, scientific, and highly specialized professions
3	Technical professions
4	Clerical support workers
5	Skilled workers in commerce and services
6	Craftspeople, skilled workers, and farmers
7	Plant operators, machinery workers, and drivers
8	Elementary occupations
9	Armed forces

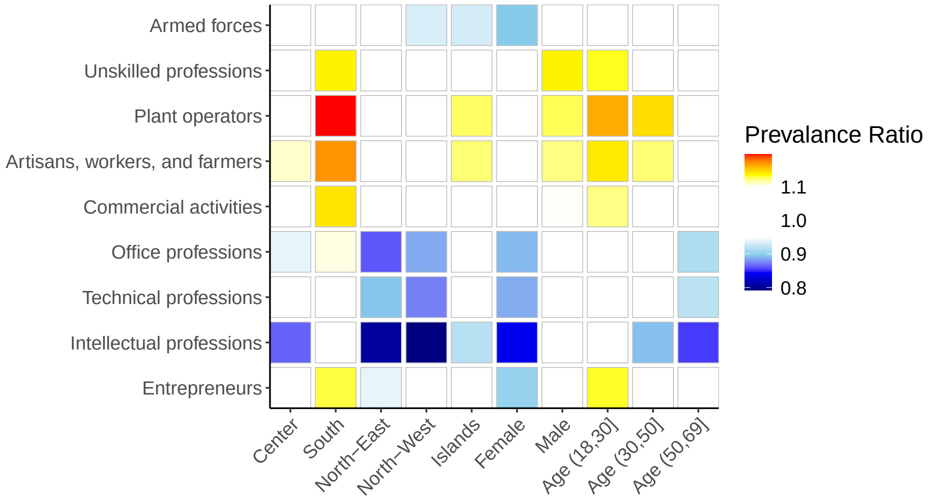

Figure S1 Prevalence ratio between the group-specific prevalence and the overall population prevalence of exhibiting at least one risky behavior by demographic variable (x-axis) and ISTAT job category (y-axis).

Appendix B | DETAILS ON SPIKE-AND-SLAB DYNAMIC PRIOR

According to Johnson and Rossell[2], who first introduced nonlocal priors, a density $f_{\text{slab}}(\beta)$ is nonlocal on a subspace $\mathcal{S}_\beta^0$ of the support $\mathcal{S}_\beta$ if, for every $\epsilon > 0$, there exists a neighborhood around β_0 within $\mathcal{S}_\beta^0$ such that $f_{\text{slab}}(\beta) < \epsilon$. In our setting, $\mathcal{S}_\beta = \mathbb{R}$ and $\beta_0 = 0$, implying that the nonlocal slab exhibits two modes away from zero.

Considering the specification of the slab $f_{\text{slab}}(\beta_{kpq}^{(t)})$ as a bimodal mixture of two Gaussians $\mathcal{N}(\mu_{-1}, \sigma_{-1})$ and $\mathcal{N}(\mu_1, \sigma_1)$, multiplied by a suitable weighted function $\omega(\beta_{kpq}^{(t)}; \xi)$ that reduces mass near the origin. We let $\rho(\beta_{kpq}^{(t-1)}) : \mathbb{R} \to (0, 1)$ denote the mixing probability and we set

$$\omega(\beta_{kpq}^{(t)}; \xi) = 1 - e^{-(\beta_{kpq}^{(t)}/\xi)^2}.$$

Let $\boldsymbol{\mu} = (\mu_{-1}, \mu_1)^\top$ and $\boldsymbol{\sigma} = (\sigma_{-1}, \sigma_1)^\top$ denote the mean and standard deviation vectors of the two Gaussian components. The resulting nonlocal slab density is

$$f_{\text{slab}}(\beta_{kpq}^{(t)}; \boldsymbol{\mu}, \boldsymbol{\sigma}, \beta_{kpq}^{(t-1)}, \xi) = \frac{\omega(\beta_{kpq}^{(t)}; \xi)}{c(\boldsymbol{\mu}, \boldsymbol{\sigma}, \beta_{kpq}^{(t-1)}, \xi)}[\{1-\rho(\beta_{kpq}^{(t-1)})\} f_N(\beta_{kpq}^{(t)}; \mu_{-1}, \sigma_{-1}^2) + \rho(\beta_{kpq}^{(t-1)}) f_N(\beta_{kpq}^{(t)}; \mu_1.\sigma_1^2)],$$

where $c(\boldsymbol{\mu}, \boldsymbol{\sigma}, \beta_{kpq}^{(t-1)}, \xi)$ is the normalizing constant.

Then we obtain

$$p(\beta_{kpq}^{(t)} | \beta_{kpq}^{(t-1)}) = \pi_0(\beta_{kpq}^{(t-1)}) f_N(\beta_{kpq}^{(t)}; 0, \sigma_0^2) + \sum_{l \in \{-1,1\}} \pi_l(\beta_{kpq}^{(t-1)}) \frac{\omega(\beta_{kpq}^{(t)}; \xi) f_N(\beta_{kpq}^{(t)}; \mu_l, \sigma_l^2)}{c(\boldsymbol{\mu}, \boldsymbol{\sigma}, \rho, \xi)},$$

with kernel functions

$$g_{-1}(\beta_{kpq}^{(t)}; \mu_{-1}, \sigma_{-1}, \xi) = \frac{\omega(\beta_{kpq}^{(t)}; \xi) f_N(\beta_{kpq}^{(t)}; \mu_{-1}, \sigma_{-1}^2)}{c(\boldsymbol{\mu}, \boldsymbol{\sigma}, \rho, \xi)},$$
$$g_1(\beta_{kpq}^{(t)}; \mu_1, \sigma_1, \xi) = \frac{\omega(\beta_{kpq}^{(t)}; \xi) f_N(\beta_{kpq}^{(t)}; \mu_1, \sigma_1^2)}{c(\boldsymbol{\mu}, \boldsymbol{\sigma}, \rho, \xi)},$$

and mixture weights

$$\pi_{-1}(\beta_{kpq}^{(t-1)}) = \{1 - \pi_0(\beta_{kpq}^{(t-1)})\}\{1 - \rho(\beta_{kpq}^{(t-1)})\},$$
$$\pi_1(\beta_{kpq}^{(t-1)}) = \{1 - \pi_0(\beta_{kpq}^{(t-1)})\} \rho(\beta_{kpq}^{(t-1)}), \quad \sum_{l=-1}^{1} \pi_l(\beta_{kpq}^{(t-1)}) = 1.$$

To model coefficient dynamics, we specify the prior mixture probabilities $\pi_l(\beta_{kpq}^{(t-1)})$ at time $t > 1$ as

$$\pi_0(\beta_{kpq}^{(t-1)}) = f_{\mathcal{N}}(\beta_{kpq}^{(t-1)}; 0, \sigma_0^2)/\varpi, \quad \pi_l(\beta_{kpq}^{(t-1)}) = g_l(\beta_{kpq}^{(t-1)}; \mu_l, \sigma_l, \xi)/\varpi,$$

for $l \in \{-1, 1\}$, with $\varpi = f_{\mathcal{N}}(\beta_{kpq}^{(t-1)}; 0, \sigma_0^2) + \sum_{l \in \{-1,1\}} g_l(\beta_{kpq}^{(t)}; \mu_l, \sigma_l, \xi)$. If $t = 1$, we set fixed hyperparameters $\pi_l(\beta_{kpq}^{(1)})$, for $l \in \{-1, 0, 1\}$. Figure 2 shows simple examples of dynamics in prior density of the entries of the coefficient matrix $\boldsymbol{B}$ as defined above.

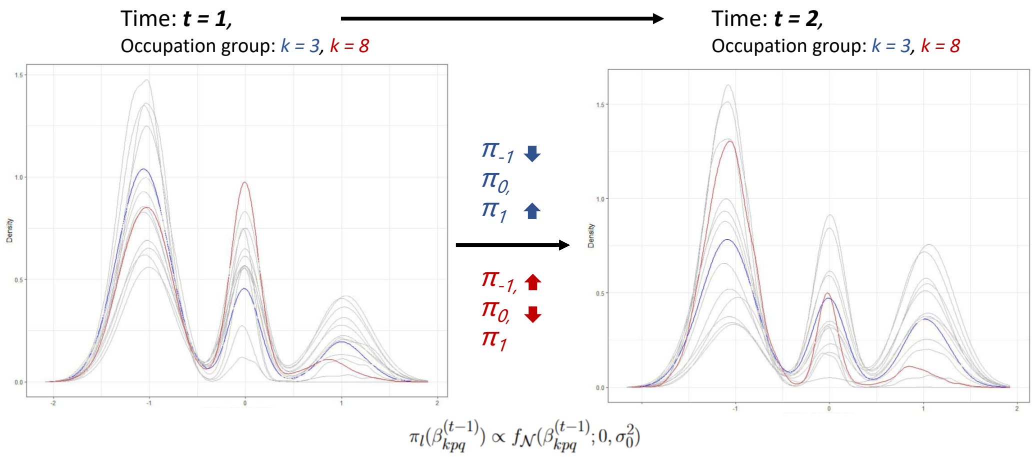

Figure S2 Example of prior density evolution for regression coefficients $\beta_{kpq}^{(1)}$–$\beta_{kpq}^{(2)}$ for two groups $k \in \{3, 8\}$.

Appendix C | COMPUTATIONAL DETAILS OF SMC ALGORITHMS

In Algorithm 2, the Metropolis-Hastings acceptance ratio α is computed in Step IIIii by considering the posterior density of $\boldsymbol{B}^{(t)}$ given $\mathcal{D}^{(1:t-1)}$ and the first n observations in period t, with $\boldsymbol{s}^{(1:t)}$ marginalized out. This quantity can be expressed as

$$p(\boldsymbol{B}_j^{(t)} | \boldsymbol{Y}^{(1:t-1)}, \boldsymbol{y}_{1:n}^{(t)} \boldsymbol{X}^{(1:t)}, \boldsymbol{W}^{(1:t)}) \propto p(\boldsymbol{B}_j^{(t)} | \mathcal{D}^{(1:t-1)}) \prod_{m=1}^{n} p(\boldsymbol{y}_m | \boldsymbol{B}_j^{(t)}, \boldsymbol{X}^{(t)}, \boldsymbol{W}^{(t)}, \boldsymbol{V}^{(t)}),$$

where the likelihood term $p(\boldsymbol{y}_m | \boldsymbol{B}_j^{(t)}, \boldsymbol{X}^{(t)}, \boldsymbol{W}^{(t)}, \boldsymbol{V}^{(t)})$ is available in closed form as a mixture over the groups $k = 1, \dots, K$. Notably, the product of likelihood terms corresponds to the joint distribution of $(\boldsymbol{y}_1^{(t)}, \dots, \boldsymbol{y}_n^{(t)})$, where independence follows from the assumptions in Subsection 3.1.

New values of $\tilde{\boldsymbol{B}}_j^{(t)}$ (proposal) are obtained by either considering the prior distribution of $\boldsymbol{B}^{(1)}$ or by jittering the current value. The prior density $p(\boldsymbol{B}_j^{(t)} | \mathcal{D}^{(1:t-1)})$ is evaluated at the current state $\boldsymbol{B}_j^{(t)}$ using results from the previous iteration $n-1$, whereas its value at the proposal $\tilde{\boldsymbol{B}}_j^{(t)}$ is computed via numerical integration. Specifically, we approximate the integral

$$p(\tilde{\boldsymbol{B}}_j^{(t)} | \mathcal{D}^{(1:t-1)}) = \int_B p(\tilde{\boldsymbol{B}}_j^{(t)} | \boldsymbol{B}^{(t-1)}) \, \mathrm{d}p(\boldsymbol{B}_j^{(t-1)} | \mathcal{D}^{(1:t-1)})$$

using the Monte Carlo average of $p(\tilde{\boldsymbol{B}}_j^{(t)} | \boldsymbol{B}_h^{(t-1)})$ over a set of H particles $\boldsymbol{B}_h^{(t-1)}$, where $h = 1, \dots, H$. These particles are sampled from the filtering distribution $p(\boldsymbol{B}^{(t-1)} | \mathcal{D}^{(t-1)})$ at the previous iteration $t-1$ of Algorithm 1.

At each time period t, one could instead rely on MCMC-based posterior approximation schemes. In Goudie et al.[3], samples are obtained sequentially by considering

$$p(\boldsymbol{s}^{(t)}, \boldsymbol{B}^{(t)} | \mathcal{D}^{(1:t)}) \propto p(\boldsymbol{s}^{(t)}, \boldsymbol{\theta}^{(t)}, \boldsymbol{B}^{(t)} | \mathcal{D}^{(t)}, \boldsymbol{B}^{(t-1)}) p(\boldsymbol{B}^{(t-1)} | \mathcal{D}^{(1:t-1)}),$$

with $p(\boldsymbol{B}^{(t-1)} | \mathcal{D}^{(1:t-1)})$ given in particles.

Appendix D | DETAILS ON SIMULATION EXPERIMENTS AND ADDITIONAL RESULTS

To set $\boldsymbol{X}$, $\bar{\boldsymbol{B}}^{(1)}$ in each scenario of the simulation experiments, we proceed as follows: for each period t, we define the binary covariate matrices $\boldsymbol{X}^{(t)}$ with dimensions $N_t \times Q$, where the first column is set to one—allowing for the intercept coefficients—and the remaining $Q-1$ columns sampled independently from a Bernoulli distribution with success probability 0.5. The entries of the first-period coefficient matrix $\bar{\boldsymbol{B}}^{(1)}$ are initially sampled from a Gaussian distribution $\mathcal{N}(0, 2)$. To enforce exact sparsity, a

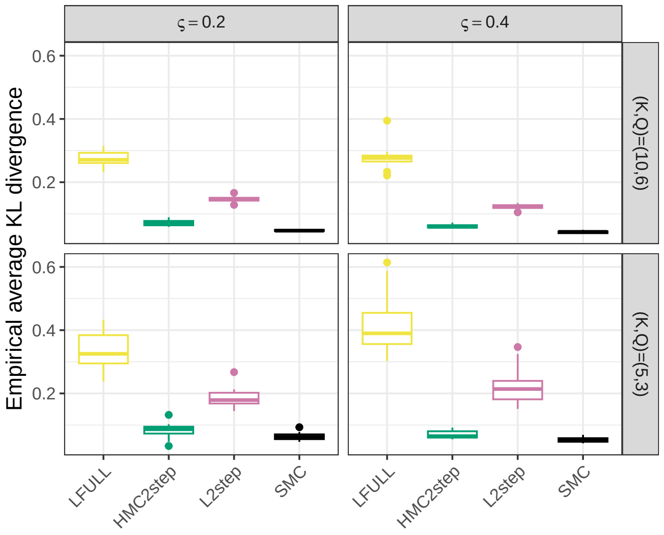

Figure S3 Boxplots of the distribution over $R = 20$ simulation replications of the average KL divergence between the benchmark predictive posterior samples provided by HMCFULL and those generated by the competing models, evaluated across varying sparsity levels ς and parameter dimensions K and Q. The number of subjects is $N_1 = 250$ and $N_2 = 300$.

thresholding step is then applied, setting entries with absolute values below the empirical quantile of level ς to zero. Temporal dynamics across periods are introduced by sampling the entries of $\bar{\boldsymbol{B}}^{(2)}$ from $\mathcal{N}(\bar{\beta}^{(1)}_{kpq}, 0.5)$ and, again, thresholding them to zero according to the ς-level empirical quantile of the drawn entries in absolute value. Group memberships $\bar{s}_n$ for the synthetic data are drawn from a categorical distribution with probabilities $\bar{\boldsymbol{\theta}}_n$, where $\bar{\boldsymbol{\theta}}_n$ is sampled from a Dirichlet distribution with parameter vector $(\exp(\bar{\boldsymbol{\eta}}_n), 1)^\top$. To emulate the inference task in the motivating application, where posterior inference on $\boldsymbol{\theta}_n$ is first conducted via topic modeling, all competing algorithms in these experiments are provided with the (true) values of $\bar{\boldsymbol{\eta}}_n$ and Σ_η, treated as a given.

After setting $\boldsymbol{X}, \bar{\boldsymbol{B}}^{(1)}, \bar{\boldsymbol{B}}^{(2)}$, and $\bar{\boldsymbol{s}}$, we replicate $R = 20$ times the generation of response variables $\boldsymbol{Y}^{(1)}$ and $\boldsymbol{Y}^{(2)}$ according to the ordinal multivariate mixture model in Equation (1). For y_p with $p \in \{1, 2\}$, we set $C_p = 3$ and $\boldsymbol{\tau}_p = (-0.5, 0.5)^\top$, while for $p \in \{3, 4\}$, we set $C_p = 4$ and $\boldsymbol{\tau}_p = (-0.75, 0, 0.75)^\top$.

We specify the following prior on $\boldsymbol{B}^{(1)}, \boldsymbol{B}^{(2)}$. Each entry $\beta^{(t)}_{jkq}$ is distributed according to nonlocal spike-and-slab prior defined in Equation 5, with $(\mu_1, \mu_{-1}) = (-1.5, 1.5)$, $(\sigma_1, \sigma_0, \sigma_{-1}) = (0.75, 0.1, 0.75)$, and $\xi = 2$.

Short description of Supplement B.

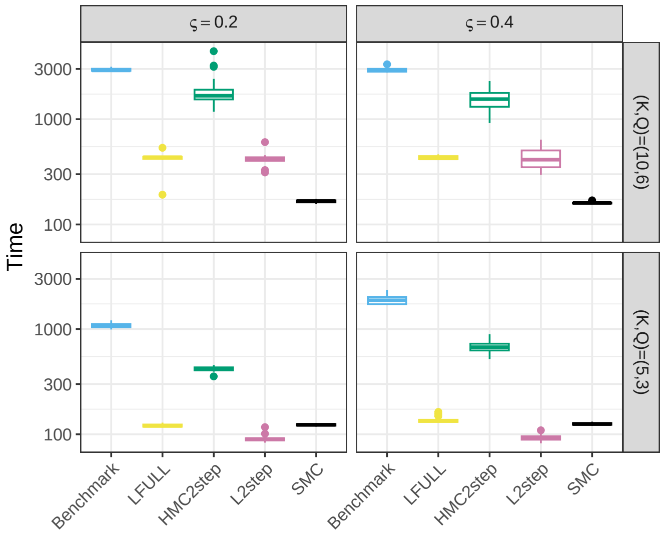

Figure S4 Boxplots of the distribution across $R = 20$ simulation replications of the time (in seconds with axis in base-10 logarithmic scale) required to generate predictive posterior samples for subjects at $t = 2$ for each competing model, evaluated across varying sparsity levels ς and parameter dimensions K and Q. The benchmark indicates the log-time distribution associated with HMCFULL. The number of subjects is $N_1 = 250$ and $N_2 = 300$.

Appendix E | ADDITIONAL APPLICATION SETTINGS AND RESULTS

We train the STM using data collected up to December 2019, i.e., $t^\star = 72$, and set $(\boldsymbol{\Lambda} = \hat{\boldsymbol{\Lambda}}_{t^\star}, \boldsymbol{\Psi}) = \hat{\boldsymbol{\Psi}}_{t^\star}$, and $\boldsymbol{\Gamma}) = \hat{\boldsymbol{\Gamma}}_{t^\star}$. Prior elicitation is completed by setting $(\mu_1.\mu_{-1}) = (-1.5, 1.5)$, $(\sigma_1, \sigma_0, \sigma_{-1}) = (0.75, 0.10, 0.75)$, and $\xi = 2$.

We employ parallel computing by running 20 parallel instances with 150 particles each, yielding a total of $J = 3000$ particles to approximate the posterior predictive distribution at each month t. Filtering a single observation requires approximately 3.5 seconds on a Windows machine equipped with an AMD EPYC 7413 24-Core Processor (2.65 GHz), using 20 cores.

Table 3 shows the representative subjects identified by the `findThoughts` function from the `stm` package[4] for each of the 30 clusters obtained from the topic model. For each cluster, the table reports the representative text $\mathbf{w}_{\tilde{n}}$, the corresponding socio-economic covariates $\mathbf{v}_{\tilde{n}}$ (i.e., economic problems and educational level), and an interpretative label summarizing these three pieces of information.

Table S3: Description of the representative subjects founded by the `findThoughts` function from the `stm` package. The label is made considering the related text and socio-economic covariates used in the STM.

Cluster	Text	Economic Problems	Edu. Level	Label
1	infermier intens ospedal professional san tecnic terap	1	High	Nurse, hospital care, higher educated, with economic problems
2	coll domest person qualific serviz tric	1	Low	Domestic helper/cleaner, lower educated, with economic problems
3	agricoltor agricoltur artigian coltiv dirett opera specializz	1	Low	Farmer and agricultural worker, lower educated, with economic problems
4	serviz addett attes attiv commercial fuoc person qualific soccors tip vigil	0	High	Security and emergency services worker, higher educated, without economic problems
5	can commerc eserciz pubblic qualific toilett	1	Low	Dog groomer, lower educated, with economic problems
6	aci adegu attual autist conducent conduttor fa impiant macchinar opera precar trasport trov veicol	1	High	Driver and vehicle operator, higher educated, with economic problems
7	san tecnic	1	Low	Healthcare worker, lower educated, with economic problems

(continues next page)

(continued)

Cluster	Text	Economic Problems	Edu. Level	Label
8	forz armat costier gurd imbarc militar mot ordin vedett	0	High	Military/coast guard, higher educated, without economic problems
9	serviz attiv commercial ipercoop person qualific	1	Low	Retail sales worker, lower educated, with economic problems
10	aliment bei chauffeur der industr qualific senn	0	Low	Food industry worker, lower educated, without economic problems
11	asil assintent nid qualific scuol univers	1	Low	Early childhood assistant, lower educated, with economic problems
12	cart legn mobil montagg qualific	0	High	Forestry and woodworking craftsperson, higher educated, without economic problems
13	assicur banc brokeragg esecut impieg lavor uffic	0	High	Insurance and banking clerk, higher educated, without economic problems
14	esecut lavor uffic amministr d professioon pubblic	0	High	Public administration clerk, higher educated, without economic problems
15	industr metalmeccan operaiain qualific	0	High	Skilled metal industry worker, higher educated, without economic problems
16	person serviz comun qualific tram	1	High	Worker in community personal services, higher educated, with economic problems
17	dirigent agricol agricoltur alta azienz imprenditor legisl ris	0	High	Agricultural entrepreneur, higher educated, without economic problems
18	tessil abbigl idustr operai qualific	1	High	Skilled textile industry worker, higher educated, with economic problems
19	x azi consulent cont ditt esecut finanziar impieg impres lavor priv pubblic revision serviz uffic	1	High	Financial and administrative office clerk, higher educated, with economic problems
20	cant ediliz geometr tecnic	0	High	Construction site technician, higher educated, without economic problems
21	acqua call center energ gas tecnic telefon	1	High	Utilities call-center, higher educated, with economic problems
22	attrezz ceram chimic stamp tecnic	1	High	Employee in the ceramics and chemical sector, higher educated, with economic problems
23	anzian assistent assunt person puliz qualific serviz	1	Low	Elderly care and cleaning staff, lower educated, with economic problems
24	elettr elettric elettron industr plc programm tecnic	0	High	Industrial electrician/technician, higher educated, without economic problems
25	elev intellettual scientif specializz profession scuol univers	0	High	Qualified educator, higher educated, without economic problems
26	ediliz mont qualific taparell zanzar	1	Low	Installation worker in construction, lower educated, with economic problems
27	attiv camer commerc commercial eserciz fast food pubblic qualific serviz	0	Low	Fast food server, lower educated, without economic problems
28	agost and april attiv bed breakfast camerier commerc commercial dicembr eserciz lugl pubblic qualific serviz stagional	1	High	Seasonal waitress, higher educated, with economic problems
29	tecnic atrezzatur cosulent lavor person serviz sicurezz	0	High	Occupational safety technician, higher educated, without economic problems
30	amministr manutenzion pubblic qualific spaz verd	1	High	Green area maintenance worker, higher educated, with economic problems

Figure 5 displays the posterior distributions of the coefficients ($\beta^{(1)}_{1k3}$, $k = 1, \ldots, K$), revealing substantial heterogeneity across latent groups in the impact of belonging to the $(50, 69]$ age class on smoking-related behavior. At $t = 1$, older workers are more likely to smoke in skilled metal industries, while the reverse holds for agricultural occupations.

Figure 6 shows the temporal trends of smoking risk probabilities for a female aged 30–50 from southern Italy. Individuals working in early childhood education and care settings display a higher propensity to smoke throughout the observed period, although in the most recent years their risk converges to the levels observed in the other two groups, characterized by higher education and, in one case, better economic status. All three job groups exhibit a rising propensity for physical inactivity during the COVID-19 pandemic.

Figure 7 shows the relative risk associated with alcohol consumption, following Figure 5 from the main manuscript. We can observe that being from the North-East increases this risk, particularly for qualified educators, agricultural entrepreneurs, and industrial electricians, characterised by high educational and economic levels. Conversely, being from the South appears to have a mainly protective effect, remembering that the baseline category corresponds to individuals from Central Italy.

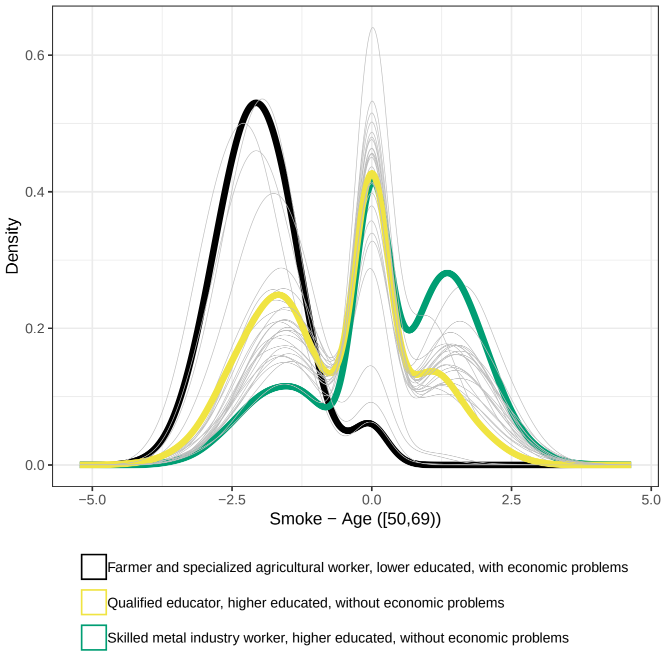

Figure S5 Posterior distributions of the coefficients $\beta^{(1)}_{1k3}$ ($k = 1, \dots, 30$) associated with the $(50, 69]$ age class and smoking-related risky behavior.

Finally, Figure 8 displays the estimated probabilities of smoking and physical inactivity across socio-demographic variables and job classes. Individuals from the South working in public administration show high levels of risk for both behaviors, particularly among younger age groups. Overall, being from the South (colored in yellow) appears to act as a risk factor for unhealthy habits such as smoking and physical inactivity across different job groups.

Figure 9 display the posterior distributions of the coefficient $\beta^{(120)}_{4k3}$, for $k = 1, \dots, 30$, associated with the effect of the $(50, 69]$ age class on physical inactivity for two profiles sharing the same occupational sector and ISTAT classification but differing in their free-text job description.

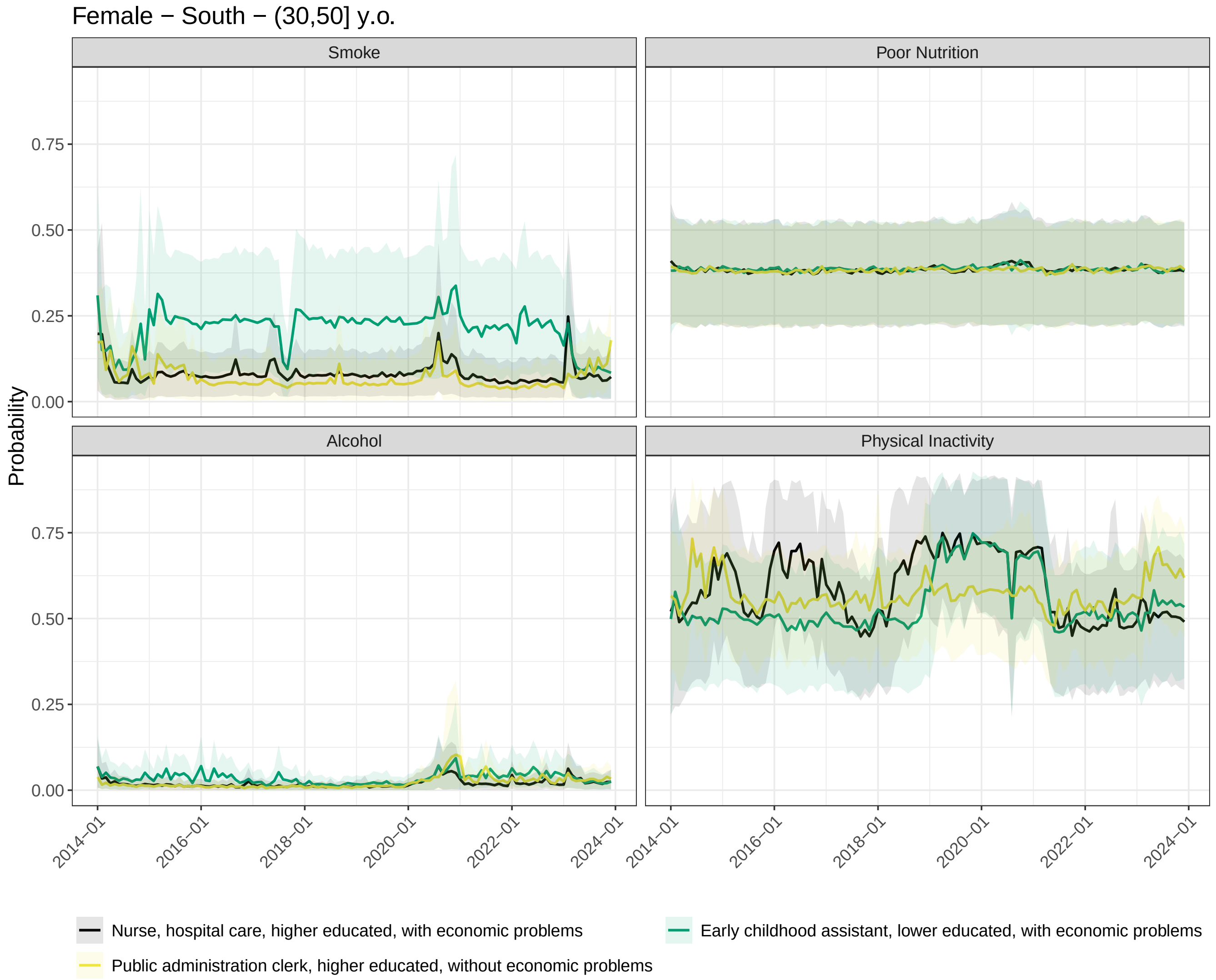

Figure S6 Estimated risk probabilities by year for each SNAP behavior, for a hypothetical 30–50-year-old female from south Italy and three latent job groups.

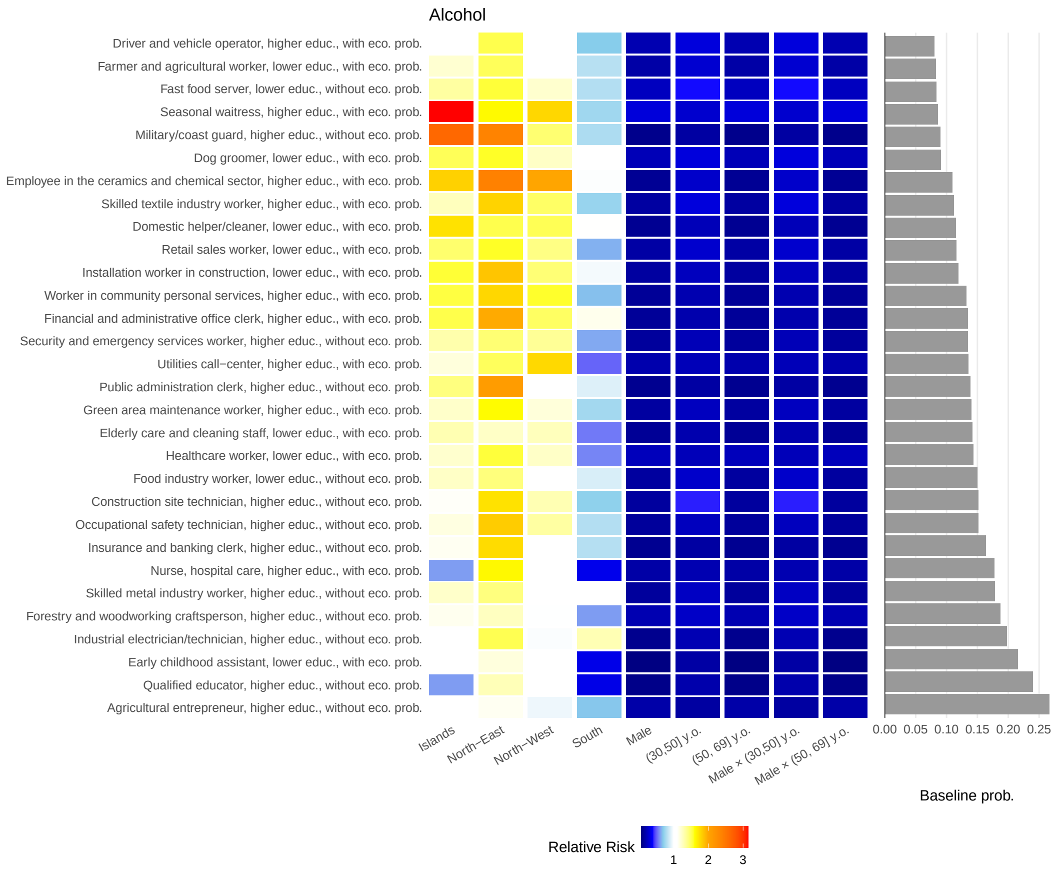

Figure S7 Alcohol relative risk for each job group and socio-demographic covariates for December 2023.

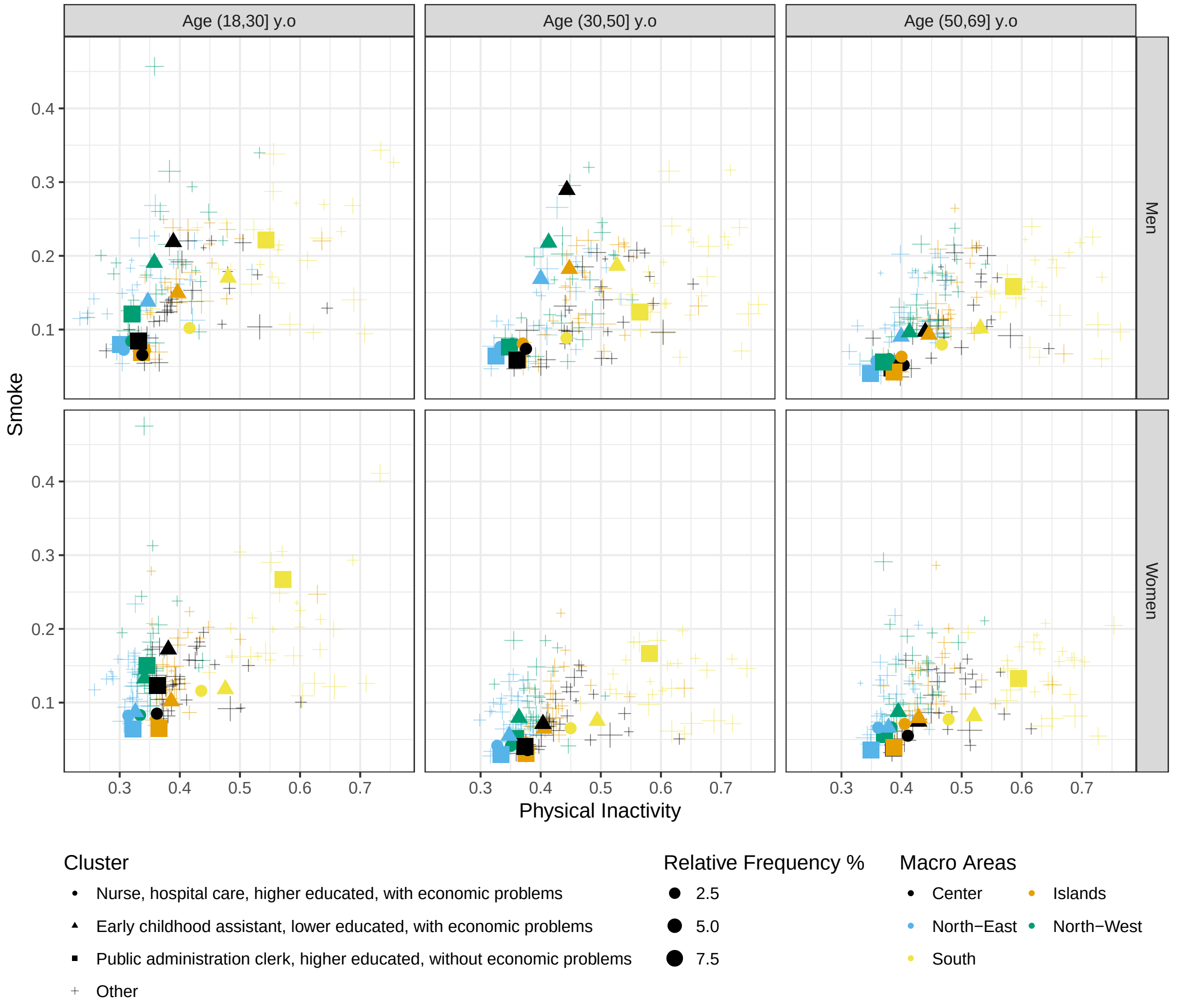

Figure S8 Estimated probabilities of engaging in risky smoke use and physical inactivity, by sex, age group, macro area, and three latent job groups.

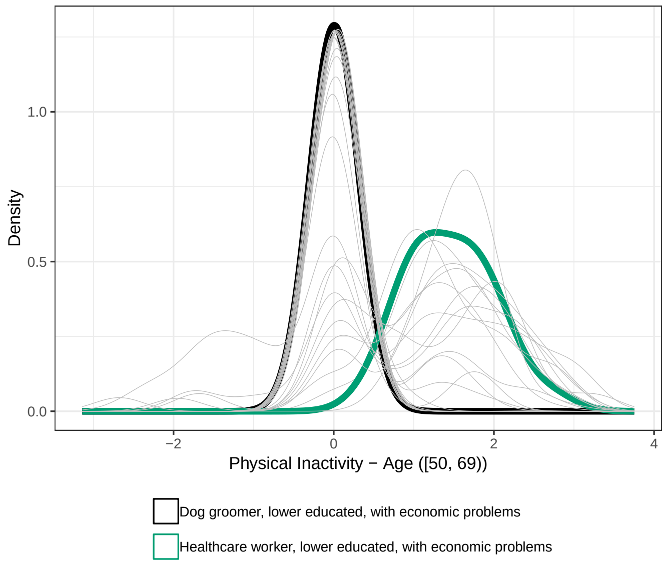

Figure S9 Posterior distributions of the coefficients $\beta_{4k3}^{(120)}$, for $k = 1, \ldots, 30$, associated with the effect of the (50, 69] age class on physical inactivity.

REFERENCES

1. Istituto Nazionale di Statistica (ISTAT) . Classificazione delle Professioni 2011 (CP2011). https://professioni.istat.it/sistemainformativoprofessioni/cp2011/; 2025. Accessed: 2025-11-10.
2. Johnson VE, Rossell D. On the use of non-local prior densities in Bayesian hypothesis tests. *Journal of the Royal Statistical Society Series B: Statistical Methodology.* 2010;72(2):143–170.
3. Goudie RJ, Presanis AM, Lunn D, De Angelis D, Wernisch L. Joining and splitting models with Markov melding. *Bayesian analysis.* 2019;14(1):81.
4. Roberts ME, Stewart BM, Tingley D. stm: An R Package for Structural Topic Models. *Journal of Statistical Software.* 2019;91(2):1–40. doi: 10.18637/jss.v091.i02
